# Fire-Safe Polymer Composites: Flame-Retardant Effect of Nanofillers

**DOI:** 10.3390/polym13040540

**Published:** 2021-02-12

**Authors:** Yukyung Kim, Sanghyuck Lee, Hyeonseok Yoon

**Affiliations:** 1 R&D Laboratory: Korea Fire Institute, 331 Jisam-ro, Giheung-gu, Yongin-si, Gyeonggi-do 17088, Korea; ykkim6025@gmail.com; 2 Department of Polymer Engineering, Graduate School, Chonnam National University, 77 Yongbong-ro, Buk-gu, Gwangju 61186, Korea; sanghyuck89@gmail.com; 3 School of Polymer Science and Engineering, Chonnam National University, 77 Yongbong-ro, Buk-gu, Gwangju 61186, Korea

**Keywords:** flame retardants, nanofillers, nanocomposites, polymers, combustion

## Abstract

Currently, polymers are competing with metals and ceramics to realize various material characteristics, including mechanical and electrical properties. However, most polymers consist of organic matter, making them vulnerable to flames and high-temperature conditions. In addition, the combustion of polymers consisting of different types of organic matter results in various gaseous hazards. Therefore, to minimize the fire damage, there has been a significant demand for developing polymers that are fire resistant or flame retardant. From this viewpoint, it is crucial to design and synthesize thermally stable polymers that are less likely to decompose into combustible gaseous species under high-temperature conditions. Flame retardants can also be introduced to further reinforce the fire performance of polymers. In this review, the combustion process of organic matter, types of flame retardants, and common flammability testing methods are reviewed. Furthermore, the latest research trends in the use of versatile nanofillers to enhance the fire performance of polymeric materials are discussed with an emphasis on their underlying action, advantages, and disadvantages.

## 1. Introduction

**Figure 1:**
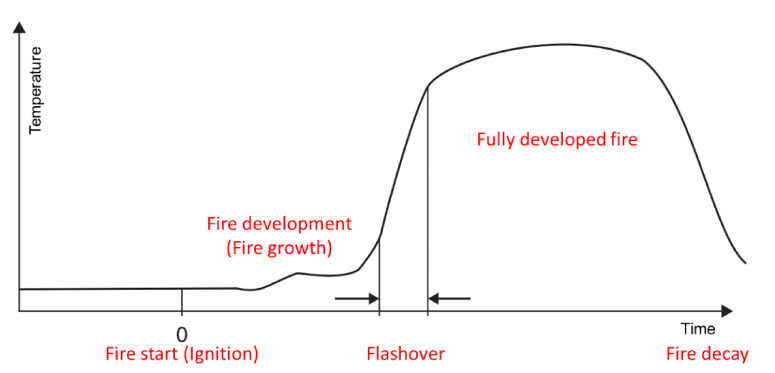
Schematic of a fire scenario. A typical fire scenario starts from ignition and fire growth, progresses to the fully developed fire, and finally reaches the fire decay stage. The fire starts and develops rapidly, and after a few minutes, it becomes severe. The generated vapor and heat initiate a flashover, which refers to the transition to total surface involvement in a fire of combustible materials. Reproduced with permission from Ref. [8]. Copyright 2011, Elsevier.

According to the statistics from the National Emergency Management Agency of South Korea during the period of 2010 to 2020, the number of large-scale fires (standard: 5 deaths, 10 casualties, and $4 million of property damage) increased six-fold from 3 to 18, and the casualties (from 45 deaths in 2010 to 232 deaths in 2019) and property damage costs (from $5 million to $330 million) increased significantly as well. These fires were mainly caused due to electrical and mechanical faults, with unknown causes also accounting for a significant proportion of these large-scale fires [1,2,3]. Based on the fire statistics for 2019, burns, smoke, and inhalation of toxic gases were the main reasons for the casualties [4,5,6,7]. Thus, fire protection becomes crucial; however, it is significantly challenging. As shown in Figure 1, a typical fire scenario includes several processes. First, ignition, which is defined as the initiation of combustion, occurs; this is followed by fire growth, which is defined as the fire development stage during which the heat release rate and fire temperature increase. During the initial stage of a fire outbreak, the fire spreads quickly, and within a few minutes, the generated smoke and heat result in “flashover.” Once the fire has reached this stage, it is difficult to control the fire [8,9,10,11,12]. Since polymer materials are used for various applications, the incorporation of functional additives to polymer materials has attracted significant research attention [13,14,15,16]. In particular, the development of flame-retardant polymer materials has attracted attention toward managing the disadvantages of heat-sensitive polymers [17,18,19,20,21,22,23]. The poisonous gases released due to combustion, which is the secondary damage caused by fire, increase the harm done to humans; therefore, developing flame retardants and flame-resistant polymer materials is still crucial [24,25,26]. The typical characteristics of a fire include the following: i) Flame spread: The size of flame and/or the time it takes for the flame to cover a defined distance from the sample; ii) dripping: The presence of flame droplets that can ignite other objects; iii) heat release: Heat generated by the combustion of a sample in the room; and iv) the opacity and toxicity of the smoke, which are important for the evacuation of people trapped in the fire.

## 2. Polymer Combustion

**Figure 2:**
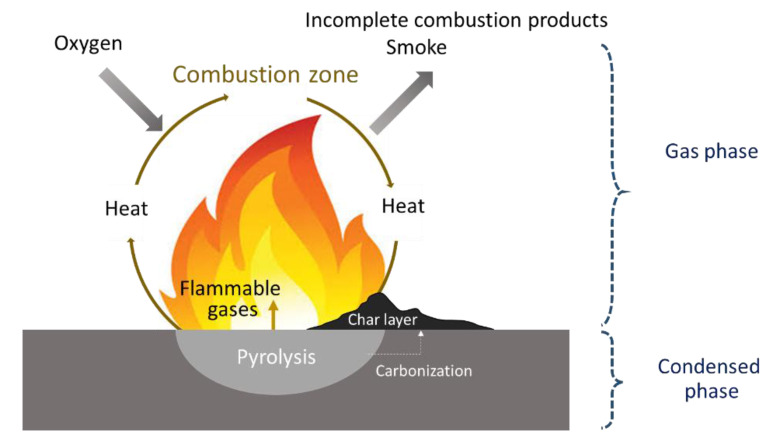
Typical combustion cycle involving a complex coupling of energy feedback from a flame to the combustible degradation products.

**Figure 3:**
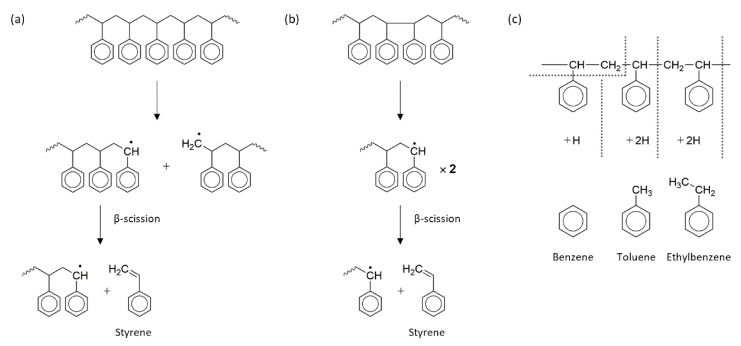
Polystyrene pyrolysis: Random-scission and end-chain β-scission process for styrene formation from (**a**) head-to-tail and (**b**) head-to-head polystyrenes (dominant at lower temperatures); (**c**) plausible processes for the generation of volatile species such as benzene, toluene, and ethylbenzene [27,28].

When exposed to sufficient heat, polymers gradually decompose and generate flammable gases that react with oxygen in ambient air to form an ignitable source. When the temperature is high enough for autoignition, ignition occurs either impulsively or at the flash point. Upon combustion, heat is released, some of which is transferred to the substrate, thereby promoting further decomposition. If there is enough heat to maintain the polymer decomposition rate such that the concentration of volatiles remains within the flammability limits, a self-sustaining combustion cycle will be established (Figure 2). Three elements, i.e., heat, oxygen, and fuel, are required to sustain the fire [8]. The heat source increases the temperature of the polymer, which depends on the strength of the heat source and inherent material properties. This temperature increase promotes the pyrolysis and formation of low-molecular-weight volatile species; a typical scheme for polymer decomposition with volatile species formation during pyrolysis is shown in Figure 3 [27]. When the volatile species combines with oxygen and the concentration reaches a critical level, the gaseous product (i.e., the mixture of fuels) ignites, and the resulting flame becomes a heat source for maintaining polymer decomposition, which is also known as the condensed phase. To suppress or reduce polymer fire, the fire cycle must be stopped by suppressing the heat, fuel, or combustion.

**Figure 4:**
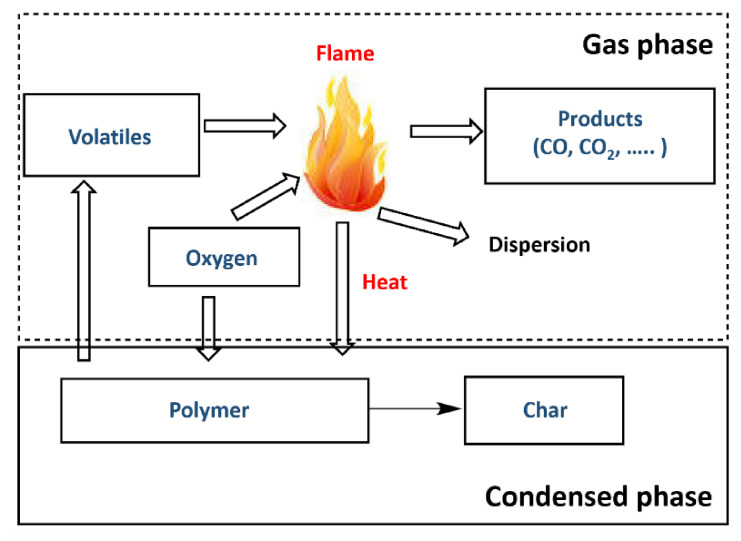
Combustion cycle of polymers. Reprinted with permission from Ref. [35]. Copyright 2016, MDPI AG.

Most natural polymer materials such as rubber and wood, are being replaced with synthetic polymers, and several organic polymer materials have been reported [15,29,30,31,32,33,34]. Synthetic polymer products, such as elastomers and plastics, can comprise one or more polymers and can contain other types of compounds, such as mineral fillers and dyes [15,35,36,37,38]. To reduce fire damage, polymer ignition delay is crucial, and it can be considered as an initial goal (Figure 4) [35]. As flame combustion is a gas-phase oxidation process, oxygen must be present in the atmosphere. Therefore, the polymer is decomposed before combustion occurs actively, since decomposition produces flammable volatile species that act as a fuel in the presence of oxygen. Table 1 shows that the combustion heats of organic synthetic polymers are greater than those of natural polymers, and the generated gas is toxic [35]. This increases the risk in the event of a fire, and thus the fire performance of organic polymers has attracted continuous attention [39,40,41,42,43,44]. Polymer flame-retarding methods include reactive and additives types, and the latter are further divided into organic and inorganic flame retardants [24,40,41,42].

**Figure 5:**
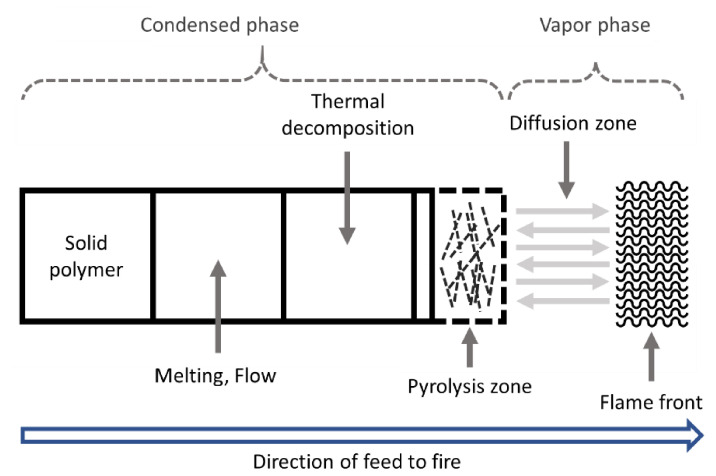
Schematic of polymer decomposition and combustion behavior. Reproduced with permission from Ref. [49]. Copyright 2012, John Wiley & Sons, Ltd.

Polymer combustion begins with heat-induced decomposition (pyrolysis) of the solid polymer, which emits volatile organic gases that mix with oxygen and result in combustion [45,46,47,48]. The heat of combustion continues the pyrolysis process that maintains a positive feedback until the cycle is broken due to the lack of heat/fuel/oxygen (Figure 5) [49]. This occurs for all the polymer materials. Thermoplastic polymers tend to lead the additional process of flame spreading or propagation, instead of generating a pyrolysis gas directly from the sample surface to the condensed phase.

**Figure 6:**
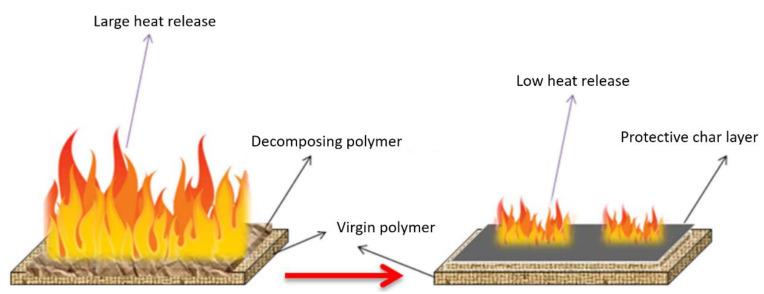
Schematic illustrating the stabilizing effect of char layer through modification of the heat release and consequent decomposition of virgin polymer during burning. Reprinted with permission from Ref. [63]. Copyright 2017, Springer-Verlag GmbH Germany.

During combustion, char-type flame retardants combine the fuel with non-pyrolytic carbon (char) to prevent fuel release and provide thermal insulation to the base polymer by forming a protective char layer [50,51,52,53,54,55,56]. In other words, the flame retardant causes charring on the polymer surface through dehydration of the flame retardant to generate double bonds in the polymer [57,58,59,60,61,62]. The carbon layer (charring) generated in this process contributes to the flame-retardant effect. Figure 6 shows an example of the charring process that can occur during the normal burning of polymers [63]. The char layer acts as a protective barrier, and during combustion, the char stability is enhanced by the decomposing polymer.

## 3. Types of Flame Retardants

**Figure 7:**
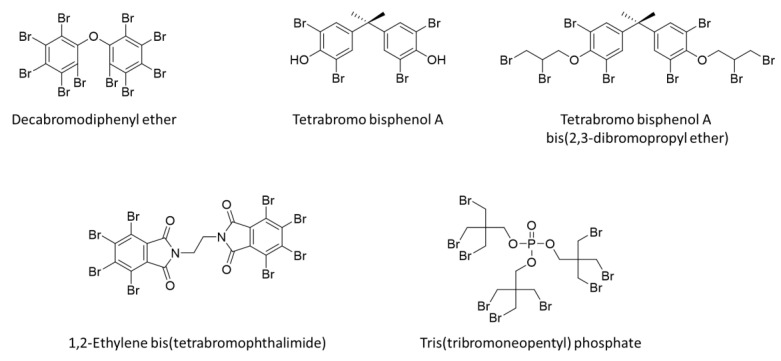
Representative chemical structures of bromine-containing flame retardants that have an inhibitory effect on combustion chemistry. Reprinted with permission from Ref. [49]. Copyright 2012, John Wiley & Sons, Ltd.

In ancient civilizations, chemicals were added to other substances for thermally activating the chemical reactions to control the flame diffusion rate or to prevent continued ignition. At that time, simple chemicals such as alum and vinegar were added to wood for providing fire safety and protection. Flame-retardant chemistry has progressed since ancient times; however, the knowledge and the basic chemicals that are available today are the same as those used decades ago. Chlorine- and bromine-based halogenated flame retardants were discovered in the 1930s, and organophosphorus flame retardants were discovered in the 1950s [64,65,66]. The first of these halogenated and phosphorus chemicals are no longer in use today, but the underlying chemical concepts are well understood and in continued use owing to their proven effectiveness over the years. The current flame retardants are products of decades of research and development and they have been tailor-made for use in certain polymers under specific circumstances [67,68,69,70,71]. Flame retardants are usually categorized into two classes: Additive (non-reactive) and reactive types, as shown in Table 2; the additive type is further divided into organic and inorganic flame retardants [72,73]. In general, additive-type methods involve the physical addition of flame-retardant components to polymers. Inorganic additives are commonly used because they are cheap, can be formulated with halogenated compounds, and can be used as fillers. Reactive-type methods are based on the chemical modification of polymers with flame retardant components, as opposed to additive-type methods [74,75,76,77,78]. According to the composition, these retardants can be classified as halogen, phosphate, melamine, and inorganic additives. Inorganic additives include aluminum hydroxide, magnesium hydroxide, zinc borate, and antimony classes [72]. Additive-type flame retardants act at high temperatures, i.e., where combustion begins, and they usually function via a deformation process without treating with the polymer. Generally, additive flame retardants are mineral fillers, hybrids, or organic compounds that can include polymeric materials. Reactive-type flame retardants are chemically incorporated into polymer chains during the polymerization or in a post-polymerization process [72]. Figure 7 shows several typical halogenated flame retardants containing bromine [49]. It should be noted that not all the organobromine compounds will provide cost-effective flame retardants. The flame-retardant material must be modified for compatibility with the polymer and should be cost-effective; furthermore, it should release bromine under the appropriate fire conditions, not too long after the polymer has begun to completely decompose or not too soon before the onset of polymer decomposition. Halogen (e.g., Br)-containing additives (RX) act by interfering with the combustion cycle, where the key combustion radicals (HO∙ and H∙) are eliminated by decomposed halogenated species, thereby effectively interfering with their oxidation. Antimony oxide is commonly used with halogenated flame retardants to yield a promotional effect. Antimony oxide works in the gas phase by facilitating the migration of halogens and antimony into the gas phase for flame retardation/inhibition. It is known that antimony oxide can be transformed to volatile antimony species, which act as effective radical species that interrupt the combustion cycle. The sequence of reactions is proposed as the steps given below.

R−Br→R·+ Br· Br· + R−H→HBr+R· H·+ Br· →HBr

$$\mathrm{HO}\cdot \;+\;\mathrm{HBr}\to {\;H}_{2}O+\mathrm{Br}\cdot \;H\cdot \;+\;\mathrm{HBr}\to {\;H}_{2}+\mathrm{Br}\cdot$$

$${\mathrm{SbBr}}_{3}+3H\cdot \;\to \mathrm{Sb}+3\mathrm{HBr}$$

Sb+HO· →SbOH

$$\mathrm{SbOH}+\mathrm{HO}\cdot \;\to \mathrm{SbO}+{\;H}_{2}O$$

**Figure 8:**
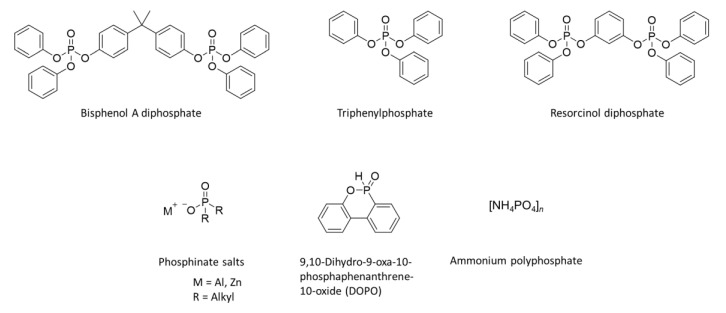
Chemical structures of representative additive-type phosphorus flame retardants, which are predominantly used in the vapor phase, and not in the condensed phase. Reprinted with permission from Ref. [79]. Copyright 2015, Springer.

Figure 8 shows several representative phosphorus compounds [79]. They are combined with other substances to usually improve the char formation or oxidative durability of the char formed by the phosphorus flame retardant. Compared to brominated retardants, there are fewer reactive versions of phosphorous flame retardants; however, only 9,10-dihydro-9-oxa-10-phosphaphenanthrene-10-oxide (DOPO) is widely used commercially [49,80,81,82,83,84,85].

### 3.1. Additive Flame Retardants

Additive flame retardants are physically mixed with polymers during the manufacturing process, in which case the flame retardants do not chemically react with the polymers. In particular, halogenated compounds containing bromine, chlorine, etc., are the most representative additive flame retardants in polymer industry because they are highly cost and performance effective [86,87,88,89,90]. Despite the related environmental and toxicity issues, halogenated compounds are still in widespread use because they are highly cost- and performance-effective. Halogen acids (HX) are representative flame retardants that scavenge active radicals (H· and OH·) and larger organic fragments (R·) to stop combustion chain reactions, resulting in flame-retardant effect [50]. Hydrobromic acid is much more effective than hydrochloric acid in this regard.

$$\begin{array}{c} H\cdot \;+\;\mathrm{HX}\;\to {\;H}_{2}+\;X\\ \mathrm{OH}\cdot \;+\;\mathrm{HX}\;\to {\;H}_{2}O\;+\;X\cdot \end{array}$$

The above equilibrium reaction is strongly influenced by temperature. Above 1000 °C, the radical scavenging activity of bromine decreases rapidly, and thus the flame-retarding properties of the halogen compound disappears. However, this remains questionable because this conclusion has not been validated experimentally. The thermal capacity of bromine, gasification heat, decomposition of flame-retardant molecules, and volume of relatively heavy halogen molecules have little dependence on temperature. Therefore, halogenated flame retardants need further testing to determine whether their activity decreases as the temperatures increases. In flames, the acid is regenerated by a hydrogen transfer reaction from species present in the flame:

X· + RH → R· + HX 

In general, bromine- or chlorine-containing compounds are used for several halogen-based flame retardants [91,92,93,94]. Particularly, bromine compounds with high bromine content and low-energy carbon–bromine covalent bonds are widely used as flame retardants in both thermoplastics and thermosets. Although the addition of a bromine compound improves the fire performance of polymer material, there is a disadvantage because it weakens the mechanical strength of the material at higher additive contents [86]. Tetrabromobisphenol A is mainly used as a bromine flame retardant owing to its low price and low thermal stability. On the other hand, chlorine flame retardants include chlorinated paraffin, chlorinated polyethylene, and aliphatic chlorine flame retardants. Chlorinated paraffin and polyethylene are inexpensive; however, they are weak in terms of thermal stability and have slightly lower flame-retardant efficiencies than that of bromine [24].

**Figure 9:**
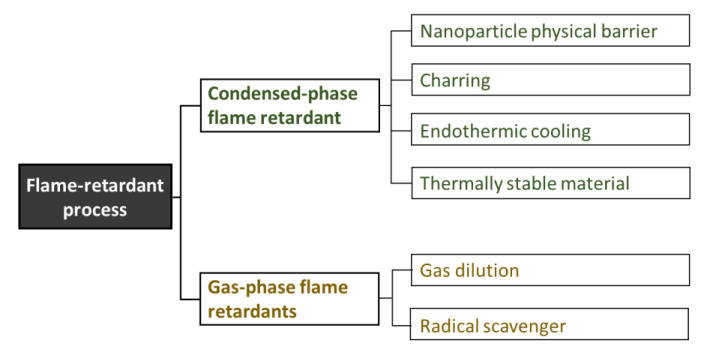
Classification of flame retardant additives based on process [95]. Reprinted with permission from Ref. [95]. Copyright 2018, Springer.

Most commercial flame-retardant additives achieve fire protection for a polymer through one or more of the following processes in the condensed- or gas-phase, as shown in Figure 9 [95]. In the condensed phase, there are numerous possible chemical reactions that can occur at high temperatures. However, scientific information on the processes and activation energies of these high-temperature reactions requires further clarification. In particular, a series of reactions in swelling-based flame-retarding systems (e.g., intumescent flame retardants) are highly sensitive to temperature, making interpretation of the processes difficult. The microstructures of polymers, including their crystallization, orientation, and phase transitions, have a significant effect on their fire performance. The migration phenomenon, which is a kind of phase transition, also plays an important role in the condensed-phase process. This is especially true for crystalline polymers such as polyolefins, polyesters, and polyamides [96,97,98,99,100]. The time when carbonaceous char forms and migrates to the polymer surface during combustion is an important variable in designing effective fire performance. The migration process is accelerated by the (i) difference in surface free energy between the molten polymer and precursor with a large carbon content, (ii) changes in temperature, and (iii) decomposition of the foaming agent. Although several studies based on the formation of carbonaceous char inhibiting the burning process of polymers have been conducted recently, there have been no clear results on the related fundamental properties and structural characteristics [37,101,102,103,104]. The widely used gas-phase process involves inert gas dilution and chemical quenching (scavenging) of active radicals. The former refers to the release of non-combustible gases during combustion, diluting the oxygen supply to the flame and/or the fuel concentration to below the flammability limit [105,106,107,108,109]. As mentioned above, several gas-phase flame retardants containing bromine and chlorine are in use, but there is an increasing need for additional non-halogenated gas-phase flame retardants. Notably, several volatile phosphorous compounds have been found to show gas-phase activities in a similar way to the halogenated flame retardants [110]. However, there is still a need for a new type of gas-phase flame retardant. These flame retardants should not contain halogens and should be cheap, stable, and non-toxic.

### 3.2. Reactive Flame Retardants

**Figure 10:**
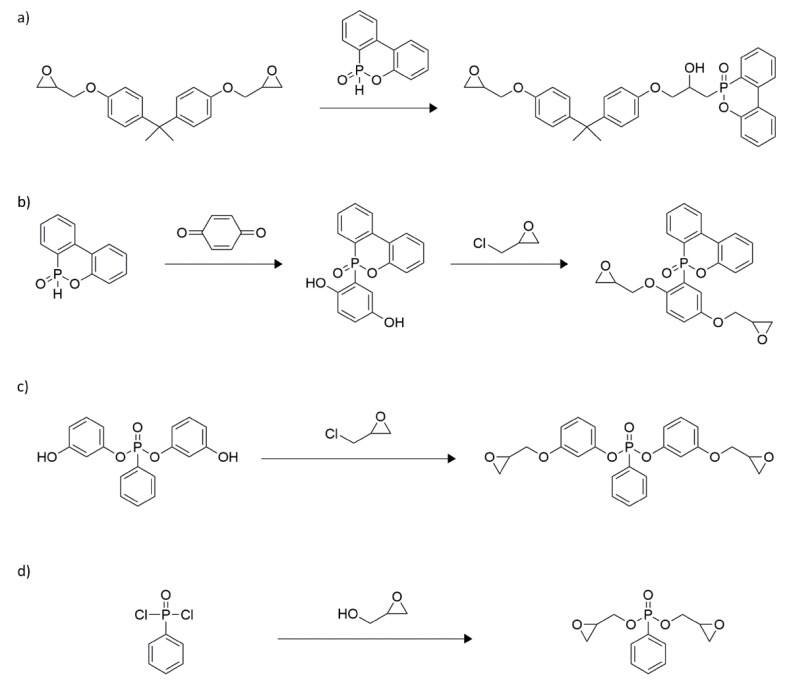
(**a**) Reaction with 9,10-dihydro-9-oxa-10-phosphaphenanthrene-10-oxide (DOPO) (phosphorus-containing flame retardant) and bisphenol A diglycidyl ether (BADGE) (epoxy resin); DOPO is the most commonly used co-monomer used to react with the oxirane ring of the epoxy component. (**b**) Reaction with DOPO modified by benzoquinone containing phenols (phosphorus-containing flame retardants) and epichlorohydrin (epoxy resin); *T*_g_ of the epoxy resin can be reduced by the co-monomers decreasing the cross-linking density. (**c**) Reaction with alcohols/phenols (phosphorus-containing flame retardants) and epichlorohydrin (epoxy resin), which is widely used as an industrial reactant; (2,5-dihydroxyphenyl) diphenyl phosphine oxide (Gly-HPO) produced by this reaction increases the limiting oxygen index (LOI) of the epoxy resin. (**d**) Reaction with (oxy)chlorides (phosphorus-containing flame retardants) and glycidyl alcohol (epoxy resin), which is an easy method to synthesize phosphorus-containing epoxy components. Reprinted with permission from Ref. [129]. Copyright 2014, Springer.

As mentioned earlier, reactive-type flame retardants involve the introduction of a monomer capable of imparting better fire resistance to a polymer, either by manufacturing a flame-retardant polymer or by introducing a reactant to a polymer, by chemically combining a flame retardant substance at the terminal or side chain [111]. Previously, flame retardant additives were used, but this method has the problem of poor commerciality with polymer materials, resulting in losses during manufacturing and use; furthermore, the mechanical properties of the polymers decrease as the amount of added flame retardant increases. To overcome these problems, a reactive flame retardant that permanently increases the fire performance can be employed by turning a part of the main chain or a pendant group of the polymer into a flame-retardant component [24]. Fire-safe polymers are prepared using this method by introducing a reactive flame retardant into the polymer in the form of monomers or polymer precursors. Therefore, reactive flame retardants are more effective in improving the fire performance of polymers than additive-type flame retardants because there are covalent bonds between the flame-retardant compound and polymer; under such conditions, the flame-retardant additive does not phase-separate or deplete. Compounds including phosphorus, nitrogen, boron, halogens, silicones, and combinations of phosphorus-nitrogen and phosphorus-silica, among others, fall into this category [112]. These compounds can be employed as chain extenders or co-monomers in polymer synthesis [112,113,114]. Among these, phosphorus-based compounds are more effective and compatible with thermoplastics resins and thermoplastic polymers. In some specific flame retardants, promotional effects occur when combinations of P-N, P-Si, and N-Si compounds are used [112,115,116,117,118,119]. In many cases, the use of reactive co-monomers is preferred since epoxy resins flame-retarded with conventional additives have poorer physical properties than unmodified ones [120,121,122,123,124,125]. Despite their shortcomings, laminated flame retardants dominate the market because most of the available reactive solutions are too complex and expensive. The most versatile method includes incorporating phosphorus-containing compounds that react easily with the hydroxyl groups of resin, resulting in a high char yield during a fire. The composition of epoxy system (e.g., the type of curing agent and presence or absence of fillers) and its application determine the necessary amount of phosphorus to meet the flammability requirements (e.g., V-0 rating, UL 94 standard). Up to 5% phosphorus is required when using anhydrous curing agents, whereas 3% is usually sufficient for amines. For laminates with a fiber content of 60%, 2% phosphorus is sufficient. Therefore, iterative optimization must be performed for all the systems. Reviews of phosphorus-containing flame retardants for epoxy resins have been published by Jain et al. [126] in 2002, Levchik et al. [127] in 2004, and Döring et al. [128] in 2010. Chemical units containing phosphorus can be introduced into the epoxy component, cross-linking agent, or both. In most cases, understanding how they behave in the gas and solid phases is necessary for further investigation. There are four main groups of synthesized phosphorus-containing epoxy components (Figure 10) [129]. The co-monomer, which is widely used to react with the oxirane ring of the epoxy component, is DOPO. By treating DOPO with bisphenol A diglycidyl ether (BADGE), the epoxy resin of 1–3% phosphorus contents was obtained (Figure 10a). A new DOPO derivative was prepared by treating with benzoquinone (DOPO-BQ). The decreased cross-linking density of co-monomers reduce *T*_g_ of the resin, which is the main drawback of their application (Figure 10b). Epichlorohydrin, which is commonly used as an industrial reactant, easily reacts with hydroxyl groups, allowing for the synthesis of diglycidyl ether of (2,5-dihydroxyphenyl) diphenyl phosphine oxide (Gly-HPO). The product of the reaction between resorcinol and phenyl phosphonic dichloride can be reacted with epichlorohydrin to form the diglycidyl ether of bis-phenoxy (3-hydroxy)phenyl phosphine oxide (Figure 10c). An efficient method to obtain phosphorus-containing epoxy components is by treating phosphorus oxychlorides with glycidyl alcohol. A series of simple epoxy components was successfully incorporated by Hergenrother et al. into an *N*,*N*,*N*′,*N*′-tetraglycidyl-4,4′-methylenedianiline–DDS system at different concentrations (Figure 10d). A phosphorus-containing diphenolic flame-retardant hardener for epoxy, 1-dopyl-1,2-(4-hydroxyphenyl)ethene was also successfully prepared by treating desoxyanisoin with DOPO in the presence of an acid catalyst [130,131].

## 4. Flammability Testing

The fire performance of a sample can be estimated by various factors such as ignitability, flame spread rate, and heat release [67,132,133,134,135]. The main tests for determining the flame behavior of a sample are limiting oxygen index (LOI), cone calorimetry, microscale combustion calorimetry (MCC), UL 94, and thermogravimetric analysis (TGA) [67]. However, a comprehensive assessment of the flame-retardant behavior of samples should involve different tests to correlate with different possible fire scenarios [136]. Each fire test represents specific conditions that cannot be generalized for other fire conditions.

### 4.1. Limiting Oxygen Index (LOI)

**Figure 11:**
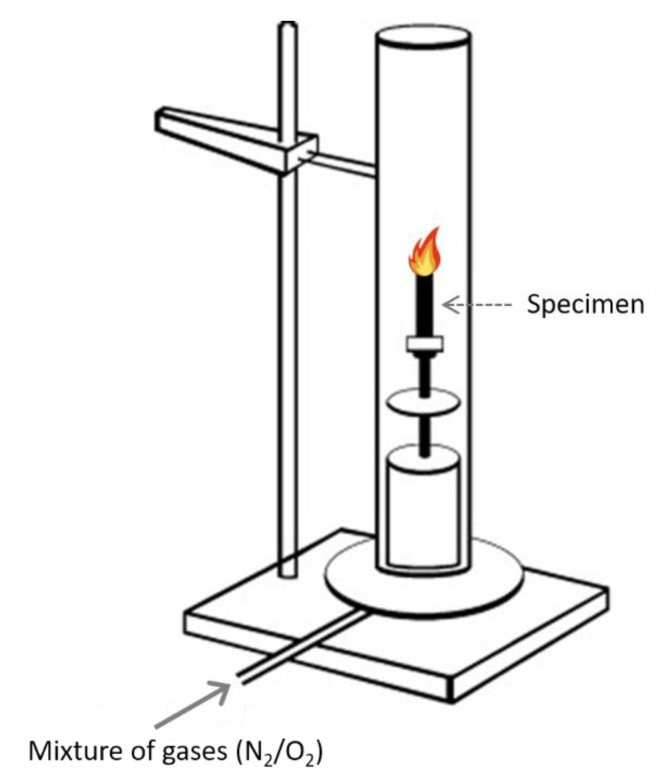
LOI test apparatus. Reprinted with permission from Ref. [137]. Copyright 2019, Elsevier.

A particularly useful laboratory test for evaluating the fire performance of samples is the LOI (ASTM D-2863) (Figure 11) [137]. LOI represents the minimum oxygen concentration in an oxygen/nitrogen mixture that can maintain the flame combustion of a material for 3 min or consumes 5 cm of sample length (ASTM D-2863 and ISO 4589) [83,138,139,140]. In accordance with ISO 4589, LOI is measured for a sample (80 × 10 × 4 mm^3^) placed vertically in the center of a glass chimney. A mixture of gases flows upstream through the chimney, passes through a glass bead layer, and becomes homogenized. After the pillars are purged for approximately 30 s, the upper part of the sample is ignited like a candle. Although this test is considered relatively unsophisticated due to the development and standardization of more advanced methods, it remains one of the most important screening and quality control methods used in the plastic industry.

Since air comprises approximately 21% oxygen by volume, materials with an LOI of less than 21% can easily burn in air. Additionally, if the LOI is more than 21%, flames can be reduced after removing the ignition source. Several researchers have suggested that substances with an LOI of greater than 28% are generally the same as flame retardants, and substances between the thresholds of 21% < LOI < 28% are materials that burn slowly [67].

Fenimore and Martin developed the LOI to establish a simple and semi-quantitative test method for evaluating the ignition and combustion behavior of different polymers [141]. LOI is a measure of the minimum oxygen concentration in an oxygen–nitrogen atmosphere required to initiate and support a flame for 3 min:

$$\mathrm{LOI}\;\left(\%\right)=\frac{{\mathrm{Volume}\;\mathrm{of}\;O}_{2}\;}{{\mathrm{Volume}\;\mathrm{of}\;O}_{2}+{\mathrm{Volume}\;\mathrm{of}\;N}_{2}}\times 100$$

Some representative LOI values for different polymers are shown in Table 3, illustrating that polypropylene and polyethylene can burn in a mixture of N_2_ and O_2_ with an oxygen volume percent less than that known for air (21%) [50]. In contrast, polytetrafluoroethylene can be placed in an atmosphere of almost pure oxygen without sustaining combustion. Despite the fair reproducibility of the LOI test for evaluating the flammability of many materials, the following limitations must be realized. (1) In real-scale fires, the oxygen access to combusting polymers is generally lower than that in LOI tests. (2) Both the air (or gas) velocity and temperature around a burning object in a real-scale fire are usually higher than those in the LOI test. (3) The LOI relies on several factors that cannot be controlled in a real fire situation, including sample geometry, sample orientation relative to the flame, air (or gas) temperature around the sample, combustion time, flow and drop of the molten polymer, formation of char or similar barriers, and the filler wicking effect. Thus, the LOI test must be supplemented with other methods for a rigorous evaluation of flame-protection systems.

### 4.2. Cone Calorimetry

**Figure 12:**
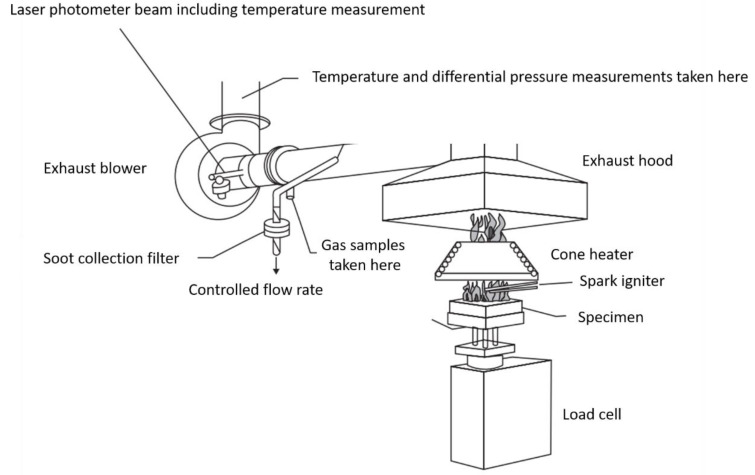
Schematic of a cone calorimeter. Reprinted with permission from Ref. [72]. Copyright 2009, Elsevier.

Cone calorimetry is one of the most useful methods for estimating the burning behavior of materials and is standardized as ASTM E-1354 and ISO 5660 (Figure 12) [72]. The method is based on measuring the decrease in oxygen concentration in the combustion gas of a sample based on a given heat flux (typically 10–100 kW m^−2^). It involves various functions such as the heat release rate (HRR), peak heat release rate (PHRR), total heat release (THR), mass loss rate (MLR), time to ignition (TTI), effective heat of combustion, and average specific extinction area (ASEA) [67]. Specimens (100 × 100 × 4 mm^3^) are placed on load cells to evaluate the mass loss evolution during the test. A conical radiant electric heater irradiates the sample uniformly from above, and combustion is triggered by electric sparks. The generated combustion gas passes through the heating cone and is captured by an exhaust duct system with a centrifugal fan and hood, which measures the gas flow; O_2_, CO, and CO_2_ concentrations; and smoke density.

**Figure 13:**
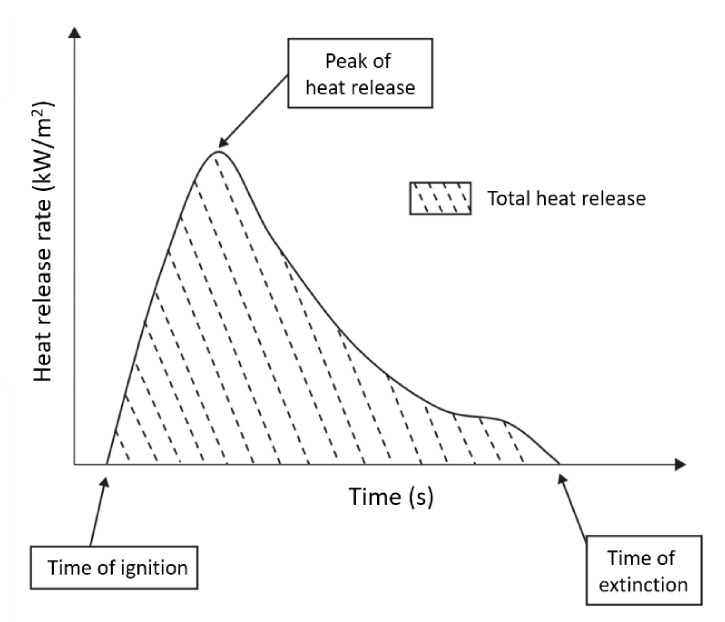
Typical cone calorimetry curve showing the time of ignition, time of extinction, peak heat release rate (PHRR), and total heat release (THR). Reprinted with permission from Ref. [8]. Copyright 2011, Elsevier.

The gas flow rate and oxygen concentration measurements are used to calculate the heat release per unit of time and surface area (HRR), which is expressed in the unit kW m^−2^. The evolution of HRR over time, especially the peak/maximum (PHRR or HRR_max_), is considered for evaluating the fire characteristics. Figure 13 shows that integration of HRR vs. time curve gives THR in kJ m^−2^ [8]. Moreover, this test allows for the characterization of ignition time, combustion or extinction time, mass loss during combustion, amounts of CO and CO_2_, and total smoke released (TSR). Table 4 lists the HRRs of several widely used polymers [142].

MCC has recently become commercially available for evaluating flammability of polymers using only 0.5–50 mg of test sample [109,143]. MCC measures the rate at which the heat of combustion of fuel gases are released by the sample during controlled pyrolysis in an inert gas stream. The instantaneous heat of combustion of the flowing gas stream is measured by oxygen consumption calorimetry.

### 4.3. UL 94

**Figure 14:**
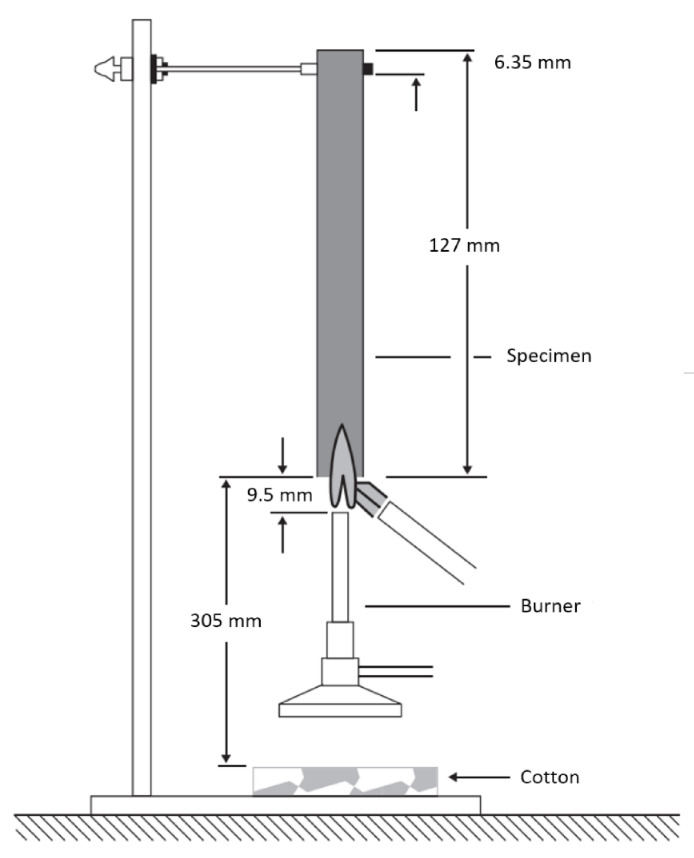
UL 94 vertical testing apparatus. Reprinted with permission from Ref. [72]. Copyright 2009, Elsevier.

UL 94 testing is also widely used in both industrial and academic research with an aim to categorize polymer materials hierarchically and meet industrial requirements [144,145,146,147,148]. Nevertheless, the information thus obtained is limited due to its basic and unfixed nature. Rather than applying the burner twice in succession for 10 s, some academic users recommend applying the burner three times for 5 s each to achieve better differentiation between the studied components. Moreover, this test appears to be less suitable for easily flowing specimens than for more cohesive materials. Vertical UL 94 specimens can be classified as V-0, V-1, or V-2 (ASTM D-3801) depending on the combustion behavior of fiber polymer (Table 5) (Figure 14) [72]. In the V-2 category, combustion stops and flaming drips are allowed within 30 s for vertical samples as long as they are not burned. A V-0 rating is given, when combustion ceases within 10 s for a vertical sample, and flaming drips are permitted unless ignition occurs [67].

### 4.4. Thermogravimetric Analysis (TGA)

TGA measures the changes in physical and chemical properties of materials as a function of temperature (ASTM E-1131 and ISO 11358). Char residues are considered as parameters obtained by TGA to analyze the thermal properties of materials at the end of the heating process, which is calculated by the weight ratio of raw materials remaining after heating and weight loss at the initial temperature (*T*_onset_) [67].

## 5. Nanoparticles as Flame-Retardant Fillers

**Figure 15:**
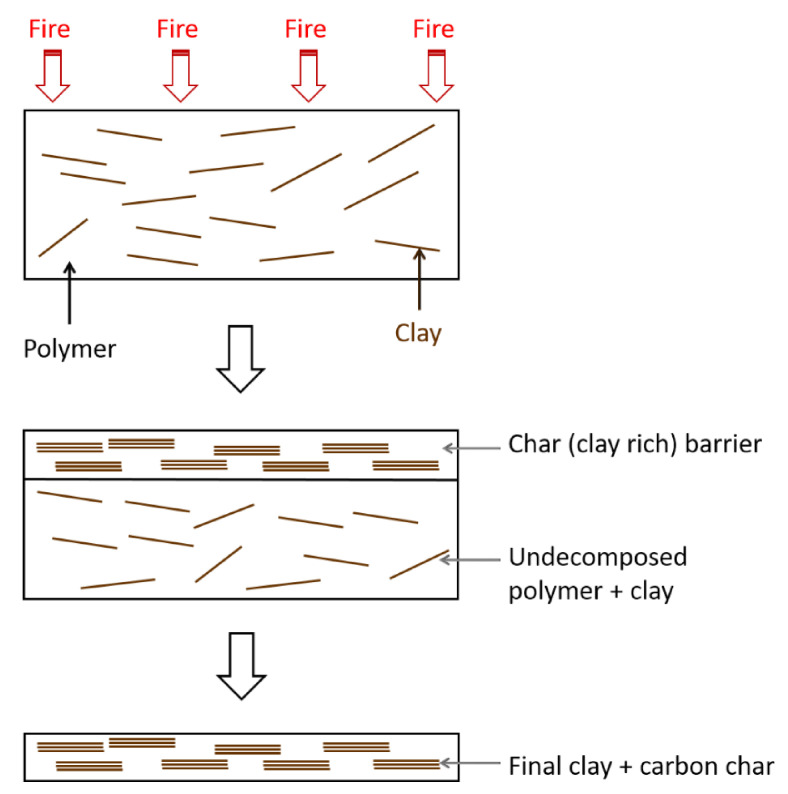
Polymer nanocomposite flame-retardant process, which decreases the HRR by char acting as a barrier. Reprinted with permission from Ref. [49]. Copyright 2012, John Wiley & Sons, Ltd.

Polymer nanocomposites are polymers reinforced with nanometer-sized particles that are finely dispersed in the polymer matrix. Natural nanoclays, i.e., layered silicates such as montmorillonite in particular, have been widely employed as fillers [136]. Recently, a variety of nanoparticles consisting of graphitic carbon, metal oxide, metal hydroxide, and metal carbide/nitride have been used as fillers to enhance the fire performance of polymers [149,150,151]. The fillers reduce the MLR of the polymer by forming a protective barrier (e.g., see the clay-rich barrier in Figure 15) inside the polymer matrix [49].

### 5.1. Nanocarbon Species

Different types of carbon fillers such as carbon black, graphite, and carbon fibers have been extensively used to improve the mechanical and electrical properties of polymer matrices [112,147,152]. Therefore, investigating the effect of carbon fillers on the fire performance of polymeric materials is crucial. Nanocarbon species, such as carbon nanotubes and graphene, have different dimensionalities, and their major chemical and physical properties can be tuned by introducing heteroatoms or functional groups. These characteristics have afforded unique nanocarbon-based fillers to improve the fire performance of polymers. Nanocarbon species have also exhibited promotional effects on fire performance upon combination with other fillers and additives [153,154].

#### 5.1.1. Single-Walled Carbon Nanotubes (SWNTs)

**Figure 16:**
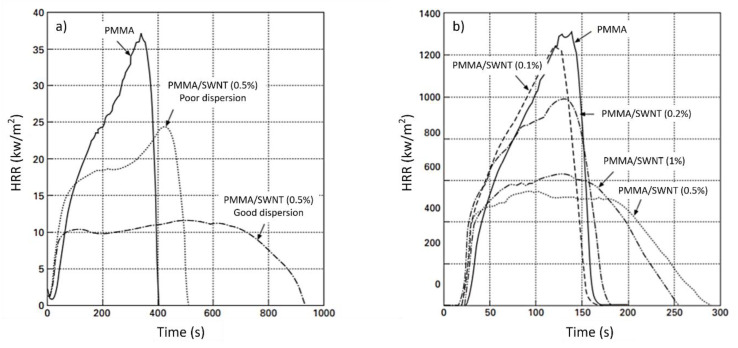
HRR of poly(methyl methacrylate) (PMMA) and PMMA/single-walled carbon nanotubes (SWNT) nanocomposites, indicating that SWNTs reduced the HRR of PMMA: (**a**) Effect of SWNT dispersion on HRR of PMMA/SWNT (0.5 wt%) nanocomposite at external radiant flux of 50 kW m^−2^. (**b**) Effects of SWNT concentration on HRR curve of PMMW/SWNT nanocomposite at 50 kW m^−2^. Reprinted with permission from Ref. [161]. Copyright 2010, Elsevier.

**Figure 17:**
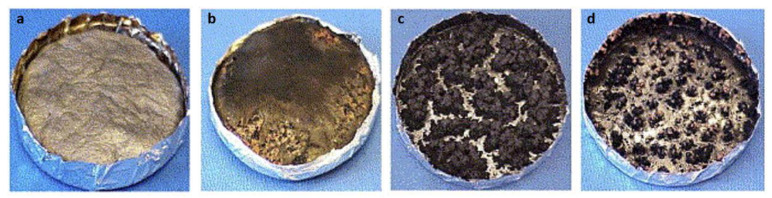
Effects of nanotube dispersion on residues of PMMA/SWNT nanocomposites (0.5%) (note: Samples were 8 and 4 mm thick for the gasification and burning test, respectively): (**a**) Nitrogen gasification residue and (**b**) burned residue of a sample with good dispersion; (**c**) nitrogen gasification residue and (**d**) burned residue of a sample with poor dispersion. Reprinted with permission from Ref. [161]. Copyright 2005, Elsevier.

**Figure 18:**
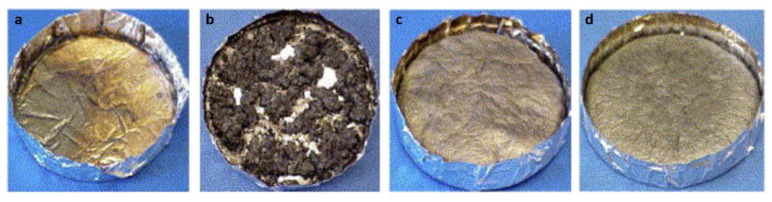
Effects of nanotube content on residues of PMMA/SWNT nanocomposites (note: The residues were obtained after gasification tests in a nitrogen atmosphere at an external radiant flux of 50 kW m^−2^): (**a**) PMMA, (**b**) PMMA/SWNTs (0.2%), (**c**) PMMA/SWNTs (0.5%), and (**d**) PMMA/SWNTs (1%). Reprinted with permission from Ref. [161]. Copyright 2005, Elsevier.

One of the most widely studied nanomaterials with respect to polymer fire performance are SWNTs [155,156,157,158,159,160]. In general, SWNTs have a positive effect on the HRR of polymers, and when nanofillers are used, the PHRR can be reduced by 50–70% as determined by cone calorimetry. In practice, this has been demonstrated by Kashiwagi et al. (2005) for a mixture of PMMA and SWNTs [161]. The mixture was prepared via the dissolution of MDF in PMMA. The nanotubes were then dispersed by bath sonication for 24 h. In the final product, for an SWNP mass content of only 0.2%, the HRR was reduced by 25%. Nanometer-sized particles, when individualized and properly dispersed in a polymer matrix, affect properties such as heat, mechanical, and fire performance. As the interfacial area between the polymer and nanofibers increases significantly, the load ratio can be markedly reduced. SWNTs are the most widely studied nanofibrous materials related to polymer fire performance and are an interesting alternative to the conventional flame retardants and nanoclays. Accurately dispersing only 0.5 wt% SWNTs in PMMA resulted in a significant reduction in liberated HRR over a much longer time span than that of neat PMMA [161]. HRR reduction of more than 50% was achieved by incorporating 0.5 wt% SWNTs (Figure 16) [161]. Figure 17 and Figure 18 show the image of the residues left behind at the end of the gasification test, and Table 6 lists the amounts of the residues collected after the gasification test. Interestingly, the PMMA nanocomposites with poor SWNT dispersion or a low SWNT content (0.2 %) formed many small, black discrete islands, unlike nanocomposites with good SWNT dispersion and higher SWNT contents. The amount of residue also depended on both the dispersion and content of the incorporated SWNT. Consequently, these data indicate that the dispersion and content of SWNTs in the polymer have significant effects on the flammability of SWNT/polymer nanocomposites.

**Figure 19:**
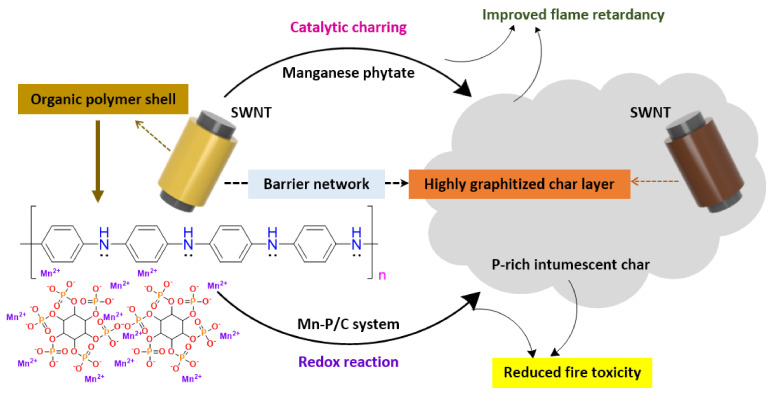
Schematic of proposed flame-retardant process for manganese pentacarbonate-dotted polyaniline-enwrapped carbon nanotubes (MPCNTs) in an epoxy composite. The dispersed state of the MPCNT particles contributes to the catalytic charring performance of the phytate structure generating a phosphorus-rich intumescent char residue and acts as a barrier network in the matrix and an efficient catalyst toward the redox reaction of the Mn-P/C system. Meanwhile, the highly graphitized char layer acts as an adhesive, making the SWNT network more close-grained. Reproduced with permission from Ref. [167]. Copyright 2018, Elsevier.

The intumescent flame-retardant process is based on the formation of a foamed cellular charred layer on a material surface above a critical temperature, which can then block the self-sustained combustion of the material at an early stage [162,163]. The density of foamed charred layer decreases with temperature; thus, it can act as an insulating shield by hindering oxygen penetration and heat transfer to the covered material. In recent years, the simultaneous reinforcement of the fire safety and mechanical performance of epoxies has been successfully achieved with several different flame-retardant systems [164,165,166]. Remarkably, manganese pentacarbonate-dotted polyaniline-enwrapped carbon nanotubes (MPCNTs) were used as refractory agents as well as epoxy reinforcing agents (Figure 19) [167]. When compared with pristine epoxy, a clear inhibitory effect on heat and CO emissions can be observed for epoxy compounds. Adding 4.0 wt% MPCNTs reduces the PHRR, THR, peak CO yield, and total CO yield. First, the well-dispersed state of MPCNTs contributes to the formation of a continuous barrier network in the matrix, which restrains the mass transfer of decomposed volatile species. In addition, the catalytic charring performance of phytate structure facilitates the formation of a phosphorus-rich intumescent char residue. Furthermore, a highly graphitized char layer is formed on the surface of nanotubes, acting as an adhesive and thus making the nanotube network more close-grained. The combination of this strengthened nanotube network with the carbonaceous char provides a compact shield for the inner material. Mn-P species in the char residue may also serve as an efficient catalyst toward redox reactions during the combustion process, which suppresses the release of toxic volatiles.

#### 5.1.2. Multi-Walled Carbon Nanotubes (MWNTs)

**Figure 20:**
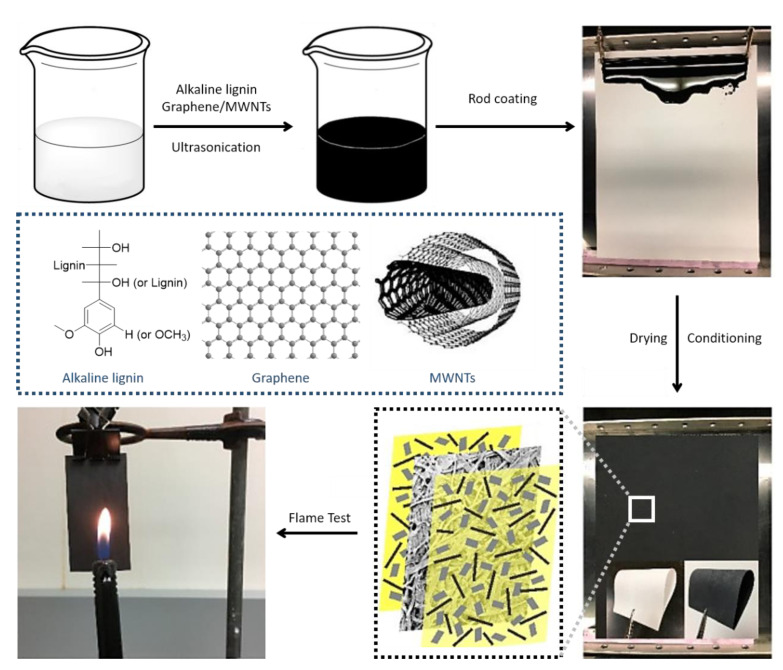
Schematic showing the fabrication of graphene nanoplatelets (GNP)/MWNT/lignin-coated paper using ultrasonication and rod coating and a flame test of the fabricated paper. Reprinted with permission from Ref. [175]. Copyright 2017, MDPI AG.

**Figure 21:**
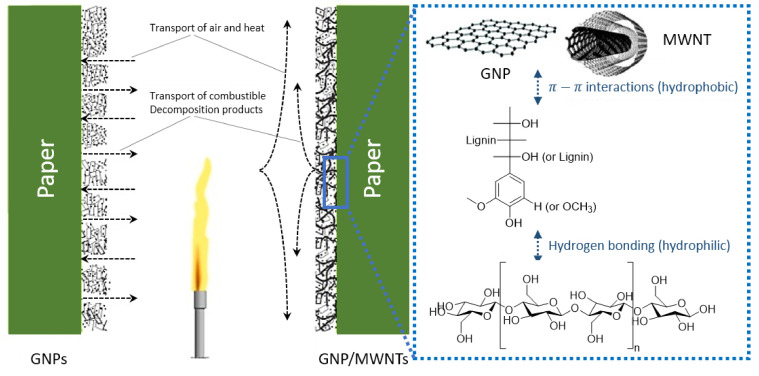
Illustration demonstrating the flame-retardant process of GNP/MWNT/lignin-coated cellulosic paper, which has multiple hydrophilic groups in the structure, allowing it to interact with cellulose via hydrogen bonding. This results in the formation of a compact layer that protects the substance from flames. Reprinted with permission from Ref. [175]. Copyright 2017, MDPI AG.

Carbon nanomaterials such as graphene nanoplatelets (GNPs) and multi-walled carbon nanotubes (MWNTs) have attracted significant attention owing to their outstanding flame-retardant characteristics in polymer composites [168,169,170,171,172]. The incorporation of these carbon nanostructures can affect the ignition delay time and reduce the HRR during the combustion of polymer composites [173,174]. Moreover, they provide other essential properties for enhanced fire protection. As shown in Figure 20 and Figure 21, GNPs and MWNTs were applied to thin films by a Meyer rod process [175]. Lightweight, flexible paper, with an increased gas constancy was obtained by coating a protective layer of carbon nanomaterials in a network structure overlapping in a random orientation [98,99,103]. The paper coated with GNP/MWNT hybrids and pre-adsorbed with lignin showed improved thermal stability and fire performance, resulting from enhanced physical barrier properties, char formation, and improved thermal management of the material. Note that GNPs, MWNTs, and lignin are all environmentally sustainable precursors [98,103].

**Figure 22:**
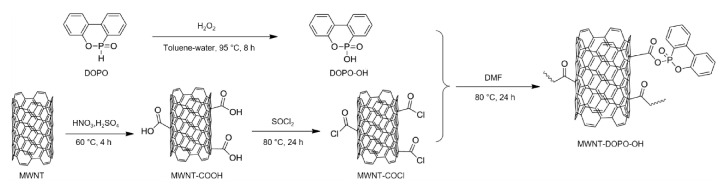
Schematic of the synthesis of MWNT–DOPO-OH. Reproduced with permission from Ref. [176]. Copyright 2018, Elsevier.

**Figure 23:**
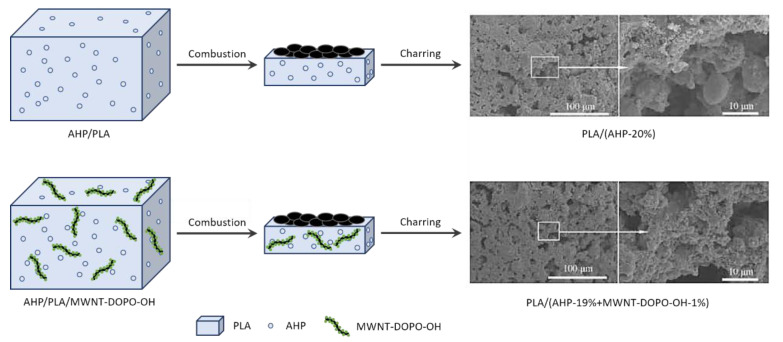
Proposed flame-retardant process for the MWNT-DOPO-OH-based polymer nanocomposites. Reproduced with permission from Ref. [176]. Copyright 2018, Elsevier.

It has been found that the incorporation of only small quantities of MWNTs can significantly enhance the mechanical and thermal properties of polymers [152,176,177,178,179,180]. Furthermore, the addition of flame-retardant-functionalized MWNTs is a highly effective way to impart favorable fire performance and mechanical properties to nanocomposites; this is because of the good dispersion of functionalized MWNTs, since the dispersion of MWNTs in a polymer matrix is considered to be a key part of improving the fire performance. Functionalized MWNTs with various organic, organometallic, or inorganic species using both non-covalent and covalent approaches improve the polymer compatibility, which makes them easier to disperse in the polymer matrix [176]. Recently, 10-hydroxy-9,10-dihydro-9-oxa-10-phosphaphenanthrene-10-oxide (DOPO-OH) was grafted onto the surfaces of MWNTs to produce DOPO-functionalized MWNTs (MWNT-DOPO-OH) by a three-step process (Figure 22) [176]. The resultant MWNT-DOPO-OH core-shell nanostructures were added into aluminum hypophosphite/poly(lactic acid) (AHP/PLA) flame-retardant systems via melt blending to enhance both the thermal and mechanical properties. AHP/PLA/MWNT-DOPO-OH nanocomposites with the promotional effect of 1 wt% MWNT-DOPO-OH and 14 wt% AHP were shown to have an improved fire performance, with a UL 94 V-0 rating and LOI of 28.6%. Furthermore, these flame-retardant nanocomposites result in a usable *T*_g_ higher than 57.8 °C and higher char yield for preventing anti-dropping. The addition of MWNT-DOPO-OH also enhanced the mechanical properties of the AHP/PLA nanocomposite (Figure 23) [176]. A cold rolling process was performed to further homogenize the constituents of the nanocomposites, which resulted in better mechanical properties even under severe plastic deformation.

**Figure 24:**
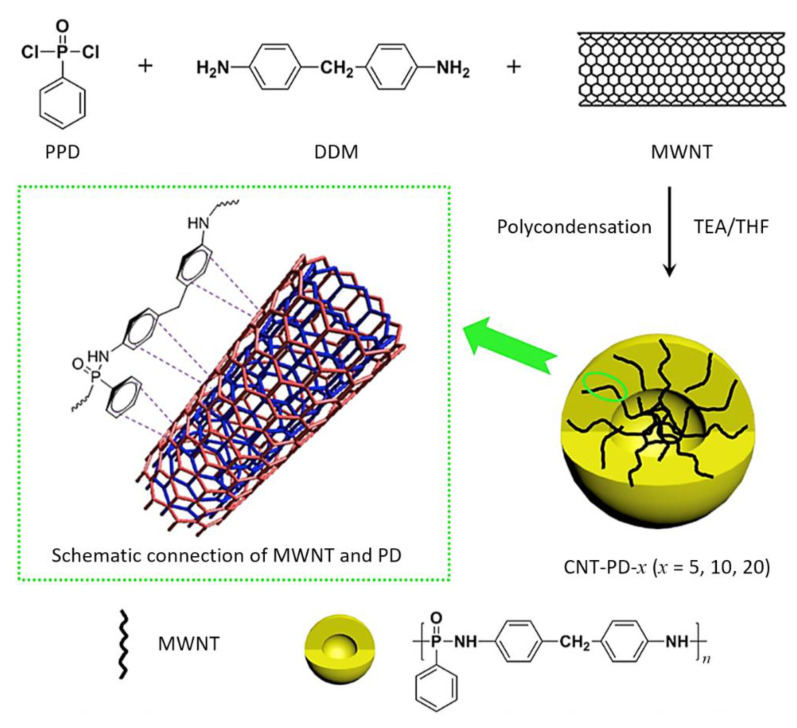
Schematic of the synthesis of MWNTs-PD-*x*. PD was wrapped in MWNTs by polycondensation. Reprinted with permission from Ref. [181]. Copyright 2016, Elsevier.

**Figure 25:**
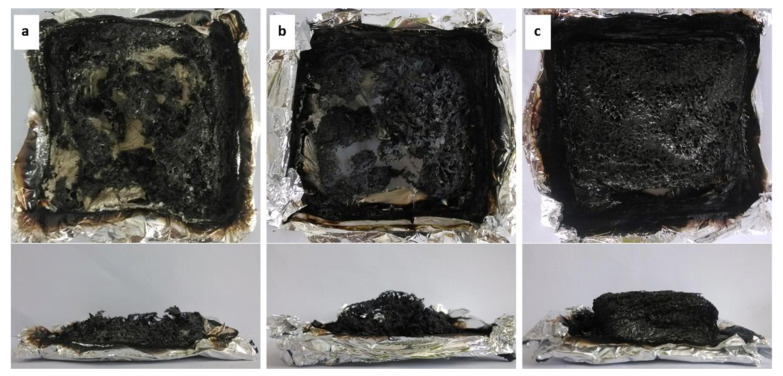
Digital images of cone calorimetry residues of (**a**) pristine epoxy with much broken char, (**b**) 2%-MWNTs/epoxy with local destruction, and (**c**) 2%-MWNTs-PD-10/epoxy with preferable intumescent char. Reprinted with permission from Ref. [181]. Copyright 2016, Elsevier.

As shown in Figure 24, the new phosphorus- and nitrogen-containing polymer-wrapped carbon nanotubes (MWNTs-PD-*x*) were readily prepared through strong π–π-stacking interaction between poly(-phenylphosphonic-4,4′-diaminodenyl-methane) (PD) and MWNT walls [181]. The polymer PD content of MWNTs-PD-*x* can be controlled by changing the supplied ratio of the polymer monomer-to-MWNTs. MWNTs-PD-*x* was introduced into an epoxy resin to improve the fire performance. When the mass fraction of MWNTs-PD-*x* (*x* = 20) was 4 wt%, the LOI reached 33.6%. Compared with MWNTs, the same addition of MWNTs-PD-*x* (*x* = 10) reduced the PHRR and THR of the epoxy resin more effectively. These results indicate that MWNTs-PD-*x* prevents the gas/condensed-phase flame, which is due to the binding action of the polymer PD and MWNTs, and contributes to the fire performance of the epoxy resin. As shown in Figure 25a, the residue of pristine epoxy sample contained only small amount of severely broken char, suggesting that the sample decomposed almost completely during combustion. The 2% MWNTs/epoxy residue in Figure 25b showed local destruction, and the expansion ratio was unclear. In contrast, as shown in Figure 25c, the residue of 2% MWNTs-PD-10/epoxy was small and complete, illustrating that the additive effectively prevented heat and mass transfer and increased the char yield [181].

**Figure 26:**
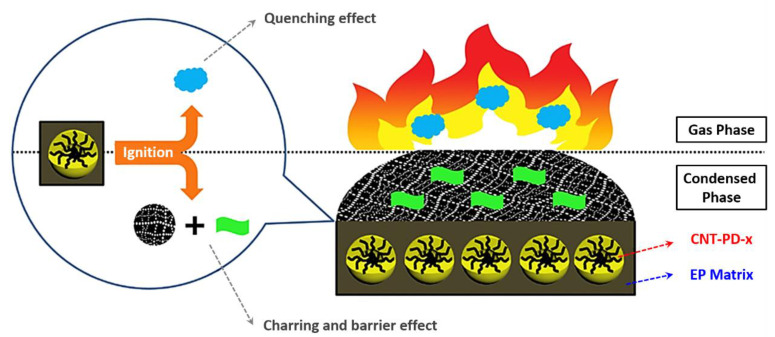
Gas/condensed-phase flame-retardant process of CNT-PD-*x*, showing that the pyrolysis of CNT-PD-*x* releases fragments that result in quenching and barrier effects. Reprinted with permission from Ref. [181]. Copyright 2016, Elsevier.

Figure 26 shows the flame-retardant process of MWNTs-PD-*x* in epoxy thermosets [181]. After the flame-retardant epoxy thermosets are ignited, the pyrolysis of MWNTs-PD-*x* releases fragments with a quenching effect on the gas phase, resulting in less complete combustion and inhibiting the intensity of the flame, thereby providing a gas-phase flame-retardant process. In contrast, the MWNTs in MWNTs-PD-*x* form a continuous-network char layer in the condensed phase. The phosphorus-nitrogen-containing polymer PD produces viscous polyphosphates and related analogs with strong dehydration properties, which not only fill the gaps of the networked char layer of MWNTs but also promote the charring of epoxy matrix. Thus, the presence of MWNTs-PD-*x* aids epoxy thermosets in forming intumescent char, which can prohibit the release of combustible volatile species from the inner matrix and heat feedback from the flame. In other words, the MWNTs-PD-*x* flame retardant has a gas/condensed-phase flame-retardant effect, wherein which the barrier effect of the intumescent char plays a key role.

One company, Bayer, has also reported polyurethane (PU)-MWNT composites as efficient flame retardants for wind turbine blades [182]. Another MWNT manufacturer, Zyvex, has also developed MWNTs and SWNTs for easy dispersion into polyurethane matrices [182]. Similar work has been conducted by Arkema (GraphiStrength), with a line of PU-MWNT master batches containing up to 45 wt% MWNTs for composite materials. These composite materials have shown several desirable properties compared with pristine PU. For example, Loss et al. found that the fatigue life of wind-resistant PU composites containing MWNTs (Baytubes C150P) increased by 248% than that of conventional systems. Köhler et al. [183] also reported that MWNT coating enhanced the thermal, mechanical, and electrical properties of PU [182].

#### 5.1.3. Graphene

Graphene and graphene oxide (GO) have exhibited promising flame-retardant performances. This is because they have a high thermal stability, strong barrier effect, and large specific surface adsorption capability, which are favorable for reducing heat and mass transfer [101,184]. Many studies have proven that graphene and its derivatives can have significant effects on the pyrolysis of polymers [36,98,99,185], as well as on their thermal conductivity, heat absorption, and dripping [184,185,186,187]. This suggests that they could be powerful flame retardants because they can ameliorate the polymer thermal stability and delay its ignition; furthermore, they can inhibit the fire from spreading and reduce the HRR [188]. A new polypropylene (PP) nanocomposite was obtained by incorporating an intumescent flame retardant (IFR), graphene, and MWNTs (purity >95%, diameter <10 nm) into a PP matrix (Table 7) [189]. The PP/IFR/MWNTs/RGO nanocomposites filled with 18 wt% IFR, 1 wt% MWNTs, and 1 wt% graphene achieved an LOI of 31.4% and UL 94 V-0 grade, and cone calorimetry showed a significant reduction in the PHR, PHRR, and ASEA of PP in terms of its combustion behavior. The TGA results indicated that the introduction of IFR, MWNTs, and graphene into PP improved the thermal stability and char yields of the nanocomposites than those of pristine PP.

**Figure 27:**
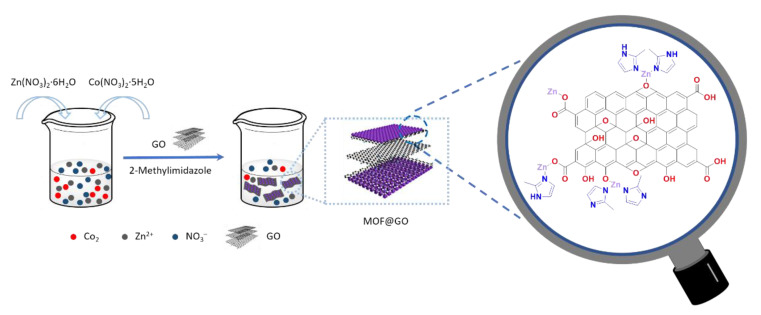
Schematic of the preparation of Zn/Co MOF@GO nanohybrids. Reprinted with permission from Ref. [195]. Copyright 2020, Elsevier.

**Figure 28:**
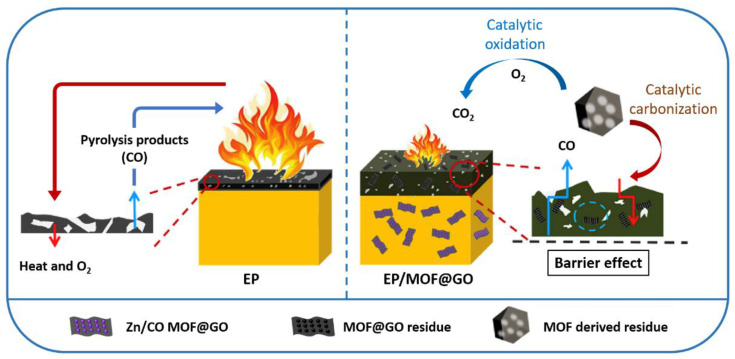
Proposed flame-retardant process of EP/MOF@GO nanohybrids. Reprinted with permission from Ref. [195]. Copyright 2020, Elsevier.

Nanohybrids of Zn/Co bimetallic metal–organic frameworks (MOFs) on GO (MOF@GO) with a sandwich structure were prepared by a simple in-situ growth method [190,191,192,193,194,195]. To achieve the homogeneous growth of MOFs on the GO layers, a new approach of adjusting the ratio of Zn and Co sources was proposed to effectively control the size and distribution of the grown MOFs (Figure 27) [195]. Uniform MOF growth on the GO layers was observed at a Zn-to-Co ratio of approximately 6:4. The optimal MOF@GO nanohybrids showed an enhanced thermal stability due to the promotional effect of the two components, and thus, they were incorporated into epoxy to reduce its fire hazards. Fire test results indicated that for an epoxy matrix with 2 wt% MOF@GO (EP/MOF@GO), the PHRR decreased by 30% and LOI increased from 23.4% to 29% than that of pristine epoxy. Moreover, the MOF@GO nanohybrids showed a decrease in the release of toxic gases; particularly, the production of CO decreased by 37% than that of pristine epoxy. The storage modulus of EP/MOF@GO also increased from 1714 to 2002 MPa. A plausible flame-retardant process proposed involves the barrier effect of layered GO, catalytic oxidation effect of transition-metal-based MOFs, and catalytic carbonization process (Figure 28) [195]. Various MOF-based hierarchical nanohybrids have shown strong potential for reducing the fire hazards of polymers while maintaining their mechanical properties [195,196,197,198]. Layered GO could act as a barrier and promote char formation in the condensed phase by inhibiting the movement of pyrolysis products into the air and permeation of oxygen and heat into the polymers. With the incorporation of MOFs, their high thermal stability can enhance the thermal stability of GO under combustion and cause the MOF@GO nanohybrids to form a more thermally stable char residue, further enhancing the effect of GO.

**Figure 29:**
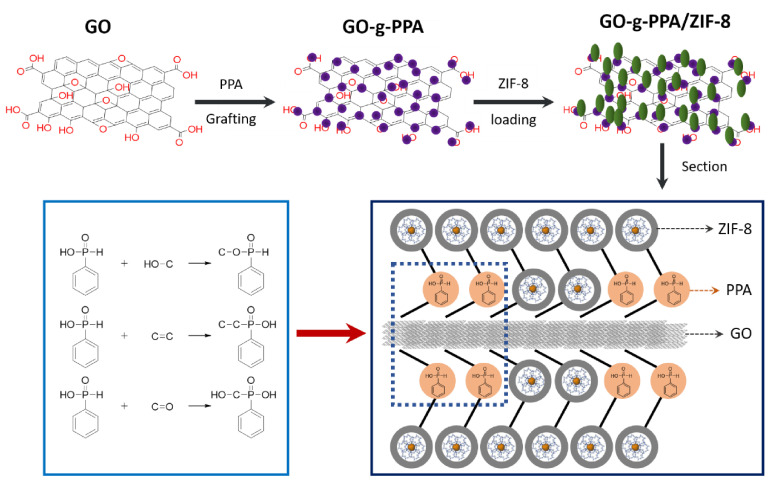
Synthesis of GPZ. Reproduced with permission from Ref. [199]. Copyright 2020, Elsevier.

Ternary hybrid (GPZ) nanoflakes consisting of GO, phenylphosphinic acid (PPA), and nano MOF (nano ZIF-8) were designed and prepared via an efficient two-step approach (Figure 29) [199]. GPZ presents a high thermal stability and good compatibility with the poly(lactic acid) (PLA) matrix. When the GPZ nanoflakes were added to PLA, the tensile strength an and toughness of PLA-4 (PLA nanocomposites with 2.0 wt% GPZ) reached 44.1 and 86.0 MPa, respectively, compared with 30.0 and 12.8 MPa for pristine PLA owing to the excellent dispersion of GPZ in the PLA matrix and reinforcing effects. The incorporation of GPZ also remarkably enhanced the fire performance of PLA, and the PHRR of PLA-4 was approximately 316.2 W g^−1^, i.e., a decrease of 39.5% than that of pure PLA (523.0 W g^−1^). The LOI of PLA-4 was 27.0%, which is an increase of approximately 31.7% than that of pure PLA (20.5%). Meanwhile, the HRR and THR of the PLA nanocomposites, as determined by cone calorimetry, were also markedly reduced [199].

#### 5.1.4. Graphitic Carbon Nitrides

Owing to its fascinating thermal and chemical stability, as well as photoelectrochemical and catalytic properties, two-dimensionally (2D) structured graphitic carbon nitride (g-C_3_N_4_), which is the most stable allotrope of carbon nitride, has recently attracted considerable attention. Analogous to graphene, g-C_3_N_4_ and its derivatives have been employed to reduce HRR, THR, and toxic gas release from polymers. g-C_3_N_4_ sheets possess surface functional groups, such as –NH_2_ and –NH, making the material easy to disperse in polymer matrices and allowing for catalytic effects. Through various promotional effects, g-C_3_N_4_ contributes to the formation of sustainable, protective char layers [200,201,202]; therefore, g-C_3_N_4_ has been combined with several other flame retardants, such as polymer nanocomposites [203,204], phosphates [205], MWNTs [206], graphene oxides [207], reduced graphene oxides [207], and metal oxides [208,209].

### 5.2. Inorganic Nanoparticles

Inorganic nanoparticles consisting of metal oxides, metal hydroxides/hydrates, and metal carbides/nitrides have been demonstrated as flame retardants [210,211,212,213,214,215]. For example, when used as a flame retardant for polymers, metal hydroxides (e.g., aluminum hydroxide (ATH) and magnesium hydroxide (MDH)) can decompose inwardly and release water at a temperature, that is higher than the polymer treatment temperature and closer to the polymer decomposition temperature. ATH and MDH are the most common inorganic flame retardants. The flame-retardant activity of MDH is highly effective up to 400 °C, above which the thermal properties of decomposition dominate. In addition, metal hydroxides (ATH and MDH) can have a catalytic effect on the combustion of resulting carbonization residue, which could account for the countercurrent observed in various flame retardation tests [72].

ATH [216]:

$$2\mathrm{Al}{\left(\mathrm{OH}\right)}_{3}+\mathrm{Heat}\;\left(180-200\;{}^{\circ}C\right)\to {\;\mathrm{Al}}_{2}O_{3}+3H_{2}O\;\left(g\right)\;↑$$

or

$${\mathrm{Al}}_{2}O_{3}\cdot 3H_{2}O+\mathrm{Heat}\;\left(180-200\;{}^{\circ}C\right)\to {\;\mathrm{Al}}_{2}O_{3}+3H_{2}O\;\left(g\right)\;↑$$

MDH [216]:

$$\mathrm{Mg}{\left(\mathrm{OH}\right)}_{2}+\mathrm{Heat}\;\left(300-320\;{}^{\circ}C\right)\to \mathrm{MgO}+{\;H}_{2}O\;\left(g\right)\;↑$$

Aluminum oxide hydrate (AlOOH or boehmite) is a common inorganic flame retardant. Boehmite decomposes at 320 °C, whereas ATH decomposes at approximately 200 °C. Therefore, boehmite can be used in engineering thermoplastics, which often results in a promotional effect in conjunction with phosphorus flame retardants (e.g., metal phosphinates) [128].

#### 5.2.1. Nanoparticulate Magnesium Hydroxide

**Figure 30:**
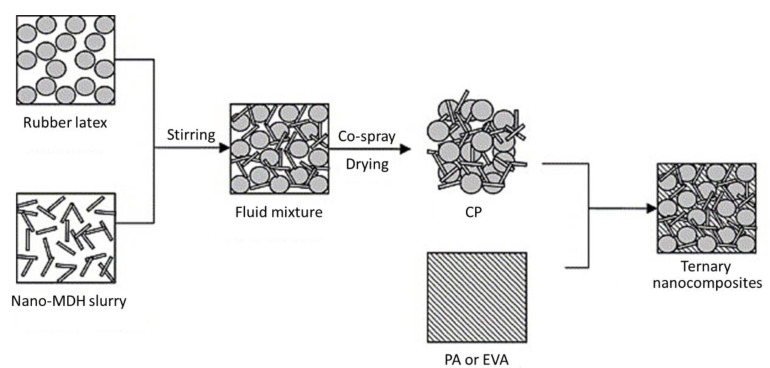
Schematic of the preparation of polyamide 6/CP_A_ and EVA/CP_B_ nanocomposites: CP_A_, CNB-UFPR/nano-MDH = 40/60 (wt%); CP_B_, NB-UFPR/nano-MDH = 40/60 (wt%). Reprinted with permission from Ref. [221]. Copyright 2006, Elsevier.

Recently, increasing environmental legislations have prohibited or limited the application of halogen-containing flame retardants to polymeric materials [217,218,219,220]. Therefore, hydrated fillers, such as ATH and MDH, have attracted interest as flame retardants for polymers. As a halogen-free flame retardant, MDH has attracted considerable attention owing to its smoke and flame suppression properties, and thermal stability, which allows for a higher processing temperature than ATH [128]. In one study, ultra-fine full-vulcanized powdered rubbers (UFPRs), which disperse uniformly in related polymer matrices owing to their unique structure and are thus used as a toughener, were employed as an additive to aid in dispersing hydrated fillers in polymer matrices (Figure 30) [221]. With the help of UFPRs, nanoparticulate MDH (nano-MDH) can be well dispersed in the polymer matrix without a surface treatment. Nano-MDH helps the elastomer disperse in the polymer matrix. A novel flame-retardant ternary nanocomposite of polymer/cross-linked rubber/nano-MDH was prepared by blending a thermoplastic polymer with a special compound powder of cross-linked rubber/nano-MDH prepared by co-spray-drying a fluid mixture of a nano-MDH slurry and irradiated rubber latex. Table 8 summarizes the compositions of three polyamide 6 nanocomposites, A0 (a control prepared without nano-MDH), A1, and A2. The cone calorimetry results showed that the new ternary nanocomposite (A1) had better fire performance than the nanocomposite (A2) obtained by a conventional process, such as a longer TTI and lower mean HRR in the initial period. It was found that the ternary nanocomposite markedly postponed the TTI, and the HRRs of samples A1 and A2 after ignition were far lower than those of sample A0 in the initial 300 s. In addition, the mean HRRs of sample A1 after ignition were 28 and 19 kW m^−2^ lower than those of sample A2 in the initial 60 and 120 s, respectively. Based on the TGA results, the onset temperature of thermal degradation for sample A1 was also higher than that of sample A2 (371 and 362 °C, respectively). Transmission electron microscopy were also used to identify the reason for this improved fire performance. A more uniform dispersion of particulate nano-MDH was observed in the new ternary nanocomposite than in the conventional one, which may account for the better fire performance.

**Figure 31:**
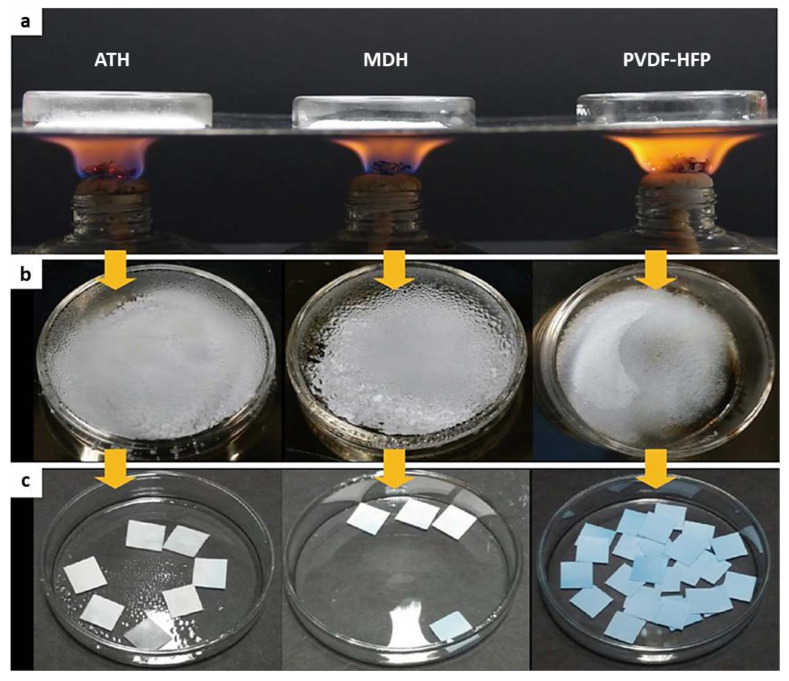
Images showing the setup for the heating experiment: (**a**) Side view and (**b**) top view. (**c**) Digital camera images of cobalt chloride (CoCl_2_) paper method for ATH and MDH. The color of the cobalt chloride paper turned from blue to white-pink, implying that the liquid droplets formed during heating were water. Reprinted with permission from Ref. [86]. Copyright 2015, Elsevier.

**Figure 32:**
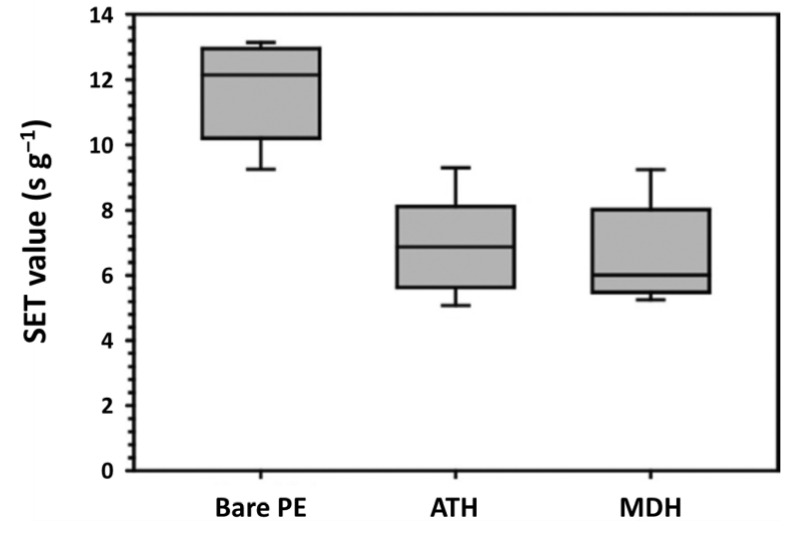
Self-extinguishing time (SET) values of separators, showing that the SETs of the aluminum hydroxide (ATH) and magnesium hydroxide (MDH) separators were much lower than that of the bare PE separator. Reprinted with permission from Ref. [86]. Copyright 2015, Elsevier.

To improve the safety of lithium-ion batteries, a composite separator was developed using ATH and MDH nanoparticles as representative hydroxides [86,222,223,224,225]. The composite separators were obtained by introducing ceramic coating layers comprising one of the chosen metal hydroxides and a poly(vinylidenefluoride-*co*-hexafluoropropylene) binder into polyethylene separators. Both metal hydroxide nanocomposite separators presented promising self-extinguishing properties, which resulted in a remarkable reduction in self-extinguishing time (SET) and helped suppress the thermal dimensional changes of the PE separators that can occur at high temperatures (Figure 31) [86]. To investigate the flame-retardant process of metal hydroxide nanoparticles, ATH and MDH were placed on a stainless-steel plate covered with a glass cap (Figure 31a) and heated on an alcohol lamp. As shown in Figure 31b, the inner surface of the glass cap facing the metal hydroxide during heating was covered with numerous liquid droplets. The liquid droplets were identified by the cobalt chloride (CoCl_2_) paper method, which is a simple, economic, and effective method based on the color shift corresponding to the change in hydration state of cobalt chloride (anhydrous cobalt chloride is blue and turns white-pink when wet) [86,226,227,228]. For both ATH and MDH, the color of cobalt chloride paper immediately changed from blue to white-pink upon absorbing the liquid droplets (Figure 31c), clearly suggesting that the liquid droplets formed during heating were water. This finding is also consistent with the flame-retardant properties of metal hydroxide nanoparticles, which release water upon heat deterioration at high temperatures. Figure 32 shows that the SETs of the composite separators were much lower than those of the bare PE separators [86]. The SETs of ATH and MDH were 50% and 42% higher, respectively, than those of the bare PE separators (12 s for bare PE, 7 s for ATH, and 6 s for MDH). Therefore, both nanocomposite separators effectively suppressed the flammability of liquid electrolyte.

#### 5.2.2. MoS_2_@TiO_2_ Nanohybrids

**Figure 33:**
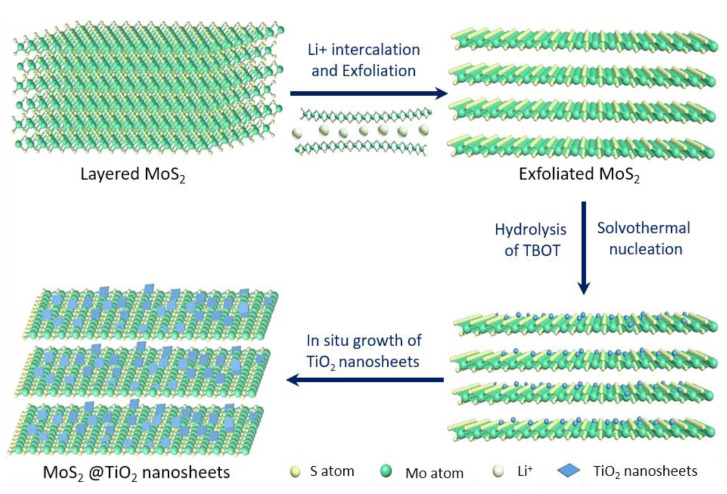
Schematic of the synthesis of hierarchical MoS_2_@TiO_2_ nanosheets. Reprinted with permission from Ref. [232]. Copyright 2019, Elsevier.

**Figure 34:**
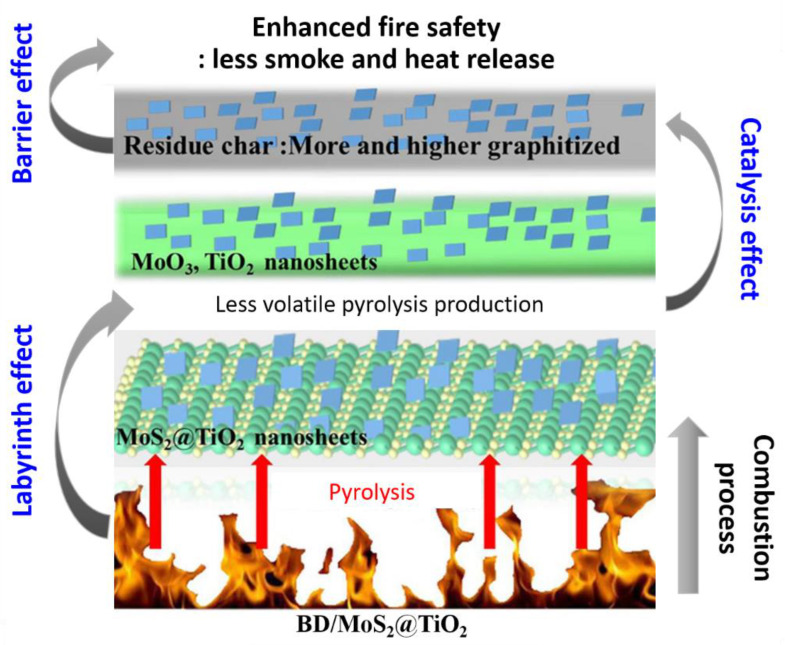
Schematic of proposed flame-retardant process for MoS_2_@TiO_2_ in BD nanocomposites. Reprinted with permission from Ref. [232]. Copyright 2019, Elsevier.

To improve the mechanical and thermal properties of bismaleimide/diallyl bisphenol A (BD), hierarchical MoS_2_@TiO_2_ nanosheets were designed and synthesized to match the high curing temperature and application temperature of BD [229,230,231]. Specifically, with a relatively low loading of 2 wt% MoS_2_@TiO_2_, the PHRR and THR of the BD nanocomposites were 314.25 kW m^–2^ and 53.58 MJ m^–2^, respectively, which were 32.5% and 15.0% less than those of pristine BD. In contrast, the TSR and yields of poisonous gases, especially carbon monoxide and nitric oxide, for BD/ MoS_2_@TiO_2_ 2.0 remarkably decreased, indicating that the fire safety was effectively enhanced. The fabrication of MoS_2_@TiO_2_ nanosheets involved two simple processes (Figure 33) [232]. First, layered bulk MoS_2_ was exfoliated into single- or few-layer sheets with the help of hydrogen gas generated by the reaction of lithium ions intercalated between the MoS_2_ layers with water [100,187]. Second, the hydrolysis of tetrabutyl titanate via a solvothermal method yielded MoS_2_@TiO_2_ nanosheets by the in situ nucleation and growth of TiO_2_ nanosheets on the surface of the exfoliated MoS_2_. While the bulk MoS_2_ displayed an opaque and dark layered structure with a size of a few micrometers, the exfoliated MoS_2_ showed a transparent, single-, or few-layer structure with an interplanar spacing of 0.61 nm and size distribution from one hundred to a few hundred nanometers. Furthermore, TiO_2_ nanosheets with a size of approximately 20 nm were uniformly grown in situ on the surface of the exfoliated MoS_2_, and overall MoS_2_@TiO_2_ nanohybrids with the (0 0 1) plane of TiO_2_ exposed were finally obtained. The layered structure of MoS_2_@TiO_2_ plays a typical barrier role in the combustion process of the BD resin, which can prevent further combustion of the internal resin due to the limited circulation of heat and oxygen. Therefore, the HRR, SPR, and production of generated gases were much lower during BD burning, thereby improving the fire safety (Figure 34) [232]. In addition, owing to the labyrinth effect of BD/MoS_2_@TiO_2_ causing lower volatile pyrolysis production and catalysis effect of MoO_3_ generated by the oxidation of MoS_2_ and the TiO_2_ nanosheets, a series of acid-catalyzed polymerizations and Diels-Alder addition reactions occurs during the combustion of the BD nanocomposites. Meanwhile, the graphitization degree of char residue of the BD/MoS_2_@TiO_2_ nanocomposites was improved and more char residue was created from the catalysis, char formation promoting effects, and physical barrier effects of MoS_2_@TiO_2_, which may also block the release of heat and pyrolysis products from the BD matrix.

#### 5.2.3. Nanoparticulate Zeolitic Imidazolate Framework-8

**Figure 35:**
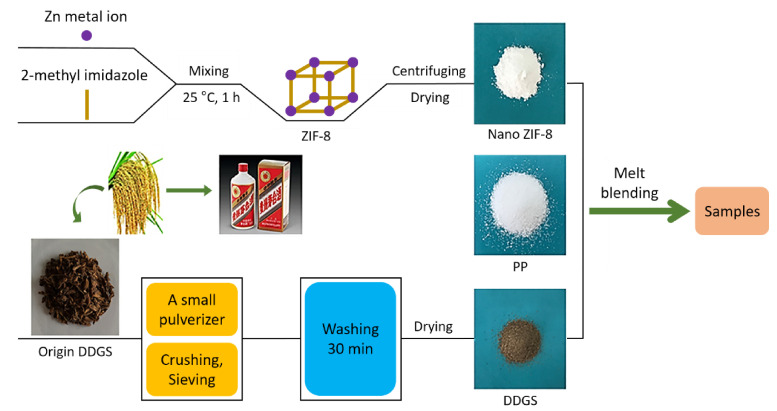
Preparation routes of polypropylene (PP) composites. Reprinted with permission from Ref. [238]. Copyright 2012, John Wiley & Sons, Ltd.

Nanoparticulate zeolitic imidazolate famework-8 (nano-ZIF-8) have been also used to develop flame retardants [233,234,235,236,237]. Recently, as shown in Figure 35 [238], a new flame retardant based on nanoparticulate zeolitic imidazolate framework-8 (nano-ZIF-8) and dried distillers grains with solubles (DDGS) was designed, without any conventional flame retardants, and blended with polypropylene (PP) to obtain an environmentally friendly flame-retardant composite [233,234,235,236,237]. The LOI of the composite containing 27 wt% DDGS and 3 wt% nano-ZIF-8 (avg. diameter of 80 ± 5 nm) was approximately 25.0%, whereas that of pure PP is 17.5%. The horizontal flame spread rate also decreased remarkably. The presence of nano-ZIF-8 and DDGS decreases the initial decomposition temperature of the composites and increases the char yield, and as a result, the combination of nano-ZIF-8 and DDGS improves the LOI and UL 94 level of the prepared composites. An improved dense char layer is formed, which can significantly enhance the fire performance of the composites.

**Figure 36:**
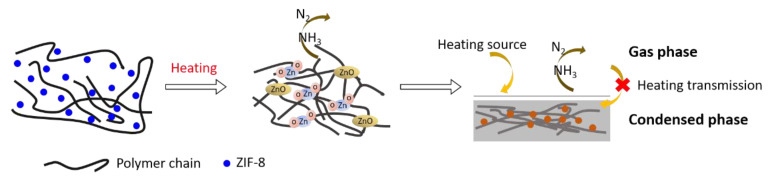
Flame-retardant process of nano-ZIF-8 particles (NZPs). Reproduced with permission from Ref. [239]. Copyright 2017, American Chemical Society.

Nano-ZIF-8 was also effective for improving the fire safety of another polymer, PLA. Nano-ZIF-8/PLA composite films were obtained by solution-blending and film-casting methods [239].The incorporation of nano-ZIF-8 enhanced the mechanical and thermal properties of PLA and influenced the polymer crystallization [40,239,240,241]. ZIF-8 is composed of Zn−O−Zn dinuclear units and imidazole units containing a large amount of nitrogen. Nitrogen-containing compounds commonly act as flame retardants, and zinc-containing compounds such as ZnO can also be employed as flame retardants or promotional flame retardants. The plausible flame-retardant process of nano-ZIF-8 is presented in Figure 36, which may involve the gas and/or solid phase [239]. First, the nano-ZIF-8 particles (NZPs) consist of imidazolate, which means that ZIF-8 contains a large amount of nitrogen. The composites thus can give off nitrogen and ammonia during burning, which releases energy and dilutes the ignitable gas. Second, the enhanced fire performance of the composites could be caused by the restricted mobility of polymer chains from the strong interactions between PLA and the NZP surface. Third, known as a class of crystalline microporous materials, NZPs show a high chemical catalytic activity. The formation of char during combustion has been attributed to dehydrogenation and cross-linking reactions catalyzed by NZPs or the decomposition products of NZPs such as ZnO. During combustion, nano-ZIF-8 causes a labyrinth effect in the polymer matrix, imparting better fire performance to the nano-ZIF-8/PLA composite.

#### 5.2.4. Modified Sb_2_O_3_ Nanoparticles

**Figure 37:**
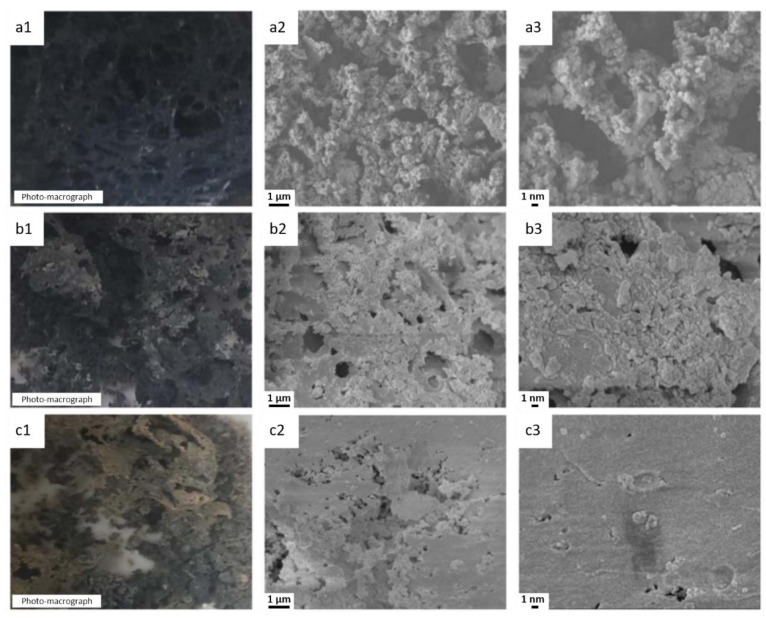
Surface morphology analysis of carbide composite materials: (**a1**–**a3**) Pure poly(butylene terephthalate) (PBT) and composites with (**b1**–**b3**) 1.5 and (**c1**–**c3**) 5 wt% nano-Sb_2_O_3_ [247]. Reproduced with permission from Ref. [248]. Copyright 2018, Taylor & Francis.

Most recent studies have focused on the promotional flame-retardant effects of micrometer-sized Sb_2_O_3_ particles and other flame retardants [242,243,244,245]. Micron-Sb_2_O_3_ and brominated IFR employed in the same system improved the char forming property of polymer matrix [246]. Upon decreasing the particle size from micrometers to nanometers, the effect of Sb_2_O_3_ on the properties of the matrix material can be enhanced, even under the same conditions. As a notable example, a nanoparticulate Sb_2_O_3_ flame retardant was obtained by high-energy ball milling and applied to poly(butylene terephthalate) (PBT) to prepare a nano-Sb_2_O_3_/PBT composite [247]. Analyses indicated that nano-Sb_2_O_3_ promoted the catalytic char formation of PBT. The samples were carbonized at 600 °C by a muffle furnace, and the char yield of the PBT composites increased gradually with increasing amount of flame-retardant additive. The highest char yield rate reached approximately 6.8% with 3.5 wt% additive, which was 12.36 times higher than that of pristine PBT. The char formation was characterized by TGA. At 600 °C, the char yield rate of pristine PBT was approximately 0.1%, which increased to approximately 5.6% and 7.6% with the addition of 1.5 and 5 wt% nano-Sb_2_O_3_ flame retardant, respectively. The results indicated that the nano-Sb_2_O_3_ flame retardant cross-linked and catalyzed PBT, promoting degradation and char formation and increasing residual carbon. Optical and SEM images of pristine PBT and composite samples carbonized with different flame retardant additive contents at 600 °C are shown in Figure 37 [247]. The char layer of the carbon residue for pristine PBT after carbonization was thin and loose, with more holes and ruptured and collapsed cells (Figure 37a). The SEM images indicated that the surface was rough, with carbonaceous particles aggregated into grape-like shapes and with more obvious holes. Therefore, pristine PBT could not form a compact and continuous char layer structure. It is likely that heat exchange occurred with the outside environment, preventing an effective barrier function. With 1.5 wt% nano-Sb_2_O_3_ additive, the number of cracks on the char layer and void sizes decreased, holes became smaller, number of holes decreased, and a number of lamellae and condensates were produced (Figure 37b). The microstructural surface roughness of the char layer was reduced, becoming dense and continuous. This kind of char layer structure could contribute to a better flame-retardant effect. When the nano-Sb_2_O_3_ additive content increased to 5 wt%, the number of holes decreased remarkably, holes became smaller, carbon residue lamella became compact and continuous, and less perforation occurred (Figure 37c). Therefore, the densification and thermal stability of the char layer were enhanced, and thus air was excluded and heat transfer was prevented. These results confirmed the outstanding flame-retardant capability of nano-Sb_2_O_3_.

#### 5.2.5. MXenes

**Figure 38:**
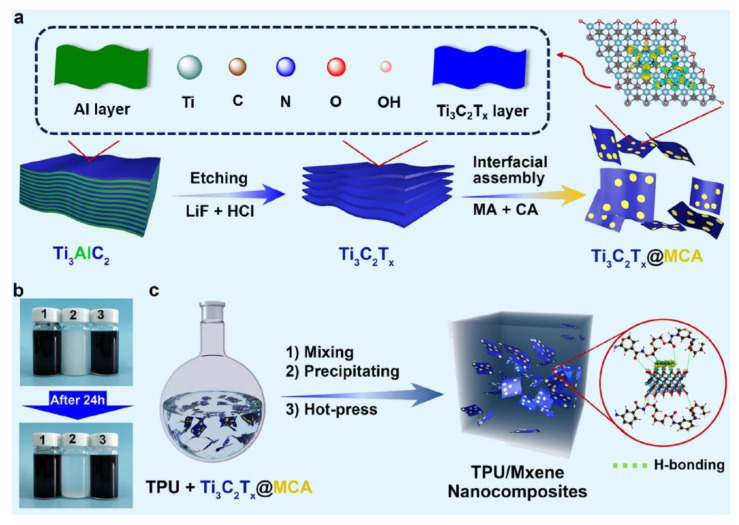
Schematics of the (**a**) synthesis of the Ti_3_C_2_T_x_@MCA nanohybrid and (**c**) preparation of TPU/Ti_3_C_2_T_x_@MCA nanocomposites; (**b**) images of (nano)additive dispersions: (1) Ti_3_C_2_T_x_ in water; (2) MCA in DMSO; and (3) Ti_3_C_2_T_x_@MCA in DMSO. Reprinted with permission from Ref. [249]. Copyright 2020, Elsevier.

**Figure 39:**
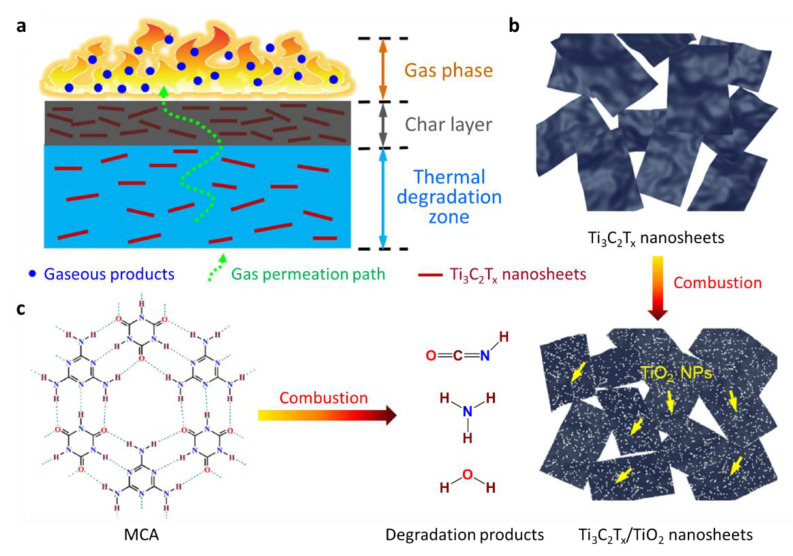
(**a**) Illustration of flame-retardant model of TPU/Ti_3_C_2_T*_x_*@MCA nanocomposites; (**b**) combustion conversion of Ti_3_C_2_T*_x_* nanosheets (TiO_2_ NPs: TiO_2_ nanoparticles); and (**c**) degradation of MCA upon combustion. Reprinted with permission from Ref. [249]. Copyright 2020, Elsevier.

A new family of 2D transition-metal carbides, carbonitrides, and nitrides, known as MXenes, has been studied for several applications, such as energy storage, electromagnetic interference shielding, and water purification. MXenes are obtained by etching “A” layer from layered MAX phases, wherein M, A, and X denote an early transition metal (such as Ti, V, Cr, or Nb), group A elements (such as Al, Si, Sn, or In), and carbon and/or nitrogen, respectively. The suffix “ene” refers to their similarity to graphene. Recently, the application of 2D layered MXenes containing carbide and/or nitride components has been extended to nanofillers for obtaining thermally stable polymer composites with good mechanical properties. It is difficult to obtain MXenes and to achieve their uniform dispersion in polymer matrices. Nevertheless, notable advances have been made by several research groups [248,249,250,251]. Representatively, Song et al. developed MXenes-based polymer nanocomposites with enhanced fire performance [249,250,251]; a notable example is the Ti_3_C_2_T_x_@MCA nanohybrid, which was manufactured by engineering the surface of titanium carbide nanosheets (Ti_3_C_2_T_x_, MXene) with melamine cyanurate (MCA) via hydrogen bonding interactions and subsequently employing in thermoplastic polyurethane (TPU)/Ti_3_C_2_T_x_@MCA nanocomposites (Figure 38). The Ti_3_C_2_T_x_ nanosheets were dispersed in an aqueous solution by first etching Ti_3_AlC_2_ in a mixture of concentrated hydrophobic acid and LiF, followed by exfoliating the layered Ti_3_C_2_T_x_ into thin nanosheets under ultrasonication. Then, the Ti_3_C_2_T_x_@MCA nanohybrid was obtained via in situ supramolecular assembly of melamine (MA) and cyanuric acid (CA) on the exfoliated Ti_3_C_2_T_x_ nanosheet surfaces in dimethyl sulfoxide (DMSO). Next, TPU nanocomposites containing Ti_3_C_2_T_x_@MCA were fabricated by a co-coagulation method coupled with a compression molding technique, as shown in Figure 38c. The PHRR values of the TPU nanocomposites were measured using cone calorimeter tests. Remarkably, the PHRR of TPU nanocomposite containing 3.0 wt% Ti_3_C_2_T_x_@MCA reduced by 40% than that of pure TPU. The TPU nanocomposites showed less char residue after cone calorimeter tests despite the low PHRR values. Ti_3_C_2_T_x_ nanosheets transformed into TiO_2_ after thermal oxidation, which can catalyze the charring of TPU. The 2D Ti_3_C_2_T_x_ nanosheets function in the condensed phase via the barrier effect of nanosheets and the catalytic effect of TiO_2_ nanoparticles generated in situ on the nanosheet surfaces and edges, which could slow down the release of combustible gases into the gas zone, thereby protecting the underlying polymer material from heat and promoting charring (Figure 39). Furthermore, MCA can first decompose into various gaseous compounds and finally evolve into polymeric carbon nitride, which thermally degrades into nonflammable nitrides at elevated temperatures. These released components can dilute the concentrations of oxygen and combustible gas products derived from TPU during burning. Therefore, it was considered that Ti_3_C_2_T_x_@MCA reduces the flammability of TPU by cooperative functions in both the gas and condensed phases through interrelated chemical and physical actions.

## 6. Conclusions and Outlook

In this review, we covered the flame-retardant combustion processes and recent flame-retardant technologies for polymers. Designing flame-retardant polymers with potential for producing new fire-safe materials is essential because large fires result in the loss of human life. Despite the challenges, flame-retardant technology is improving, and the potential fire-resistant polymer materials can decrease fire risks and damage. Understanding the basic processes of fire resistance and researching new flame-retardant processes is crucial as it is directly connected to the safety of citizens and fire prevention. In recent years, flame-retardant performance test methods such as the upgraded cone calorimeter have made it easier to analyze toxic combustion gases in microscale units, which is expected to facilitate research on flame retardants. Active gas-phase flame retardants are being studied more intensively because they can circumvent the environmental issues of halogenated flame retardants.

**Figure 40:**
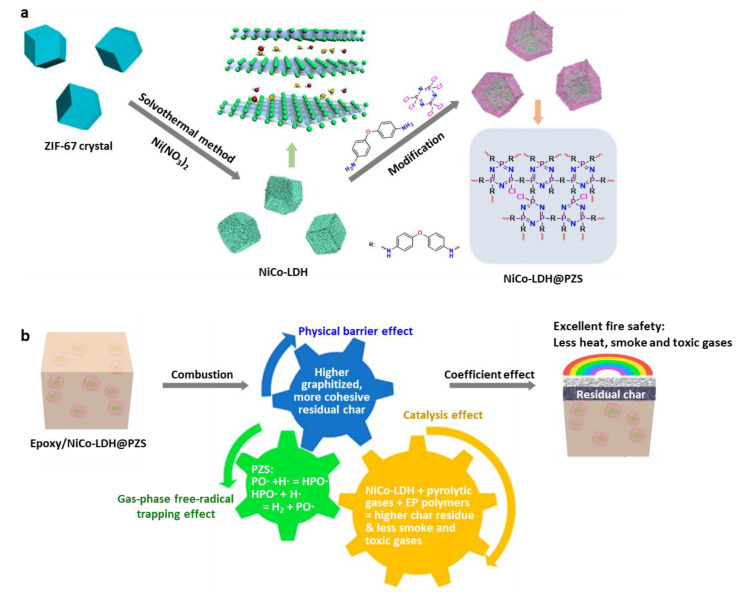
Schematics of the (**a**) synthetic route and (**b**) possible flame-retardant process of NiCo-LDH@polyphosphazene. Reprinted with permission from Ref. [164]. Copyright 2019, ACS.

In contrast, along with improving the mechanical properties, the incorporation of nanofillers into polymer matrices has been widely studied to improve the fire performance of polymeric materials. Appropriate nanofillers can result in improved fire performance and significantly elevate the smoke suppression effect of polymers. Some graphitic carbon and inorganic nanoparticles incorporated into polymer matrices in a controlled fashion have been demonstrated as capable of enhancing the polymer fire performance (Table 9). Specifically, one-dimensional SWNTs and MWNTs form network-like protective layers during combustion, which shield the surrounding/underlying polymer matrix from external radiation and heat feedback from flames. Strong nanoparticle–polymer interactions increase the viscosity of polymer melt, which can increase the nanoparticle concentration and improve the fire performance. However, in the case of SWNTs, there are limited studies showing that SWNT/polymer composite materials have effective fire performance. In contrast, several studies have employed MWNTs for enhancing the fire performance of polymers, as the interacting MWNTs result in the formation of a compact layer that protects the substance from flames. The important factor is the dispersion of SWNTs and MWNTs in the polymers because this determines the flammability effect of the polymer nanocomposite. Additionally, 2D graphene affords a strong barrier effect in polymer matrices, resulting in a high thermal stability. Graphene reduces heat release and mass transfer, as well as delays the fire ignition time, because it can promote the formation of a dense, continuous char layer during the decomposition process, which can act as a barrier to prevent heat transfer from the heat source. Furthermore, 2D layered inorganic materials such as layered double hydroxides (LDHs) have exhibited similar barrier properties [252]. One of the latest trends in developing new flame-retardant systems is combining more than two flame-retardant components. A representative example of combining different types of flame retardants is shown in Figure 40. Similarly, several flame retardants can be combined in the nanometer regimen to create promotional effects.

As mentioned before, nanofillers themselves do not usually show outstanding fire resistance such as self-extinguishing properties, and thus, they are commonly coupled with other flame-retardant additives. For improved fire performance, it is also essential to control the dispersion of nanofillers with other additives in polymer matrices. The efficient dispersion of nanofillers enables a substantial reduction in the loading amount. Moreover, the contribution of each type of nanoparticle to the fire performance of the polymer varies and strictly depends on the chemical structure and geometry [72]. The large interfacial contact area between the nanofiller and polymer may enhance catalytic effects such as the catalysis of charring reactions or radical trapping processes. It has been found that the formation of a continuous protective layer consisting of a network of nanoparticles is a key flame-retardant process for nanofillers, where the layer appears to act as a physical shield [188]. The formation of such a network is also important in reinforcing other physical properties of polymeric materials. Furthermore, the polymer composites should meet all the requirements in terms of major properties for practical applications, which is still a challenge.

Some existing flame retardants (e.g., typically halogenated compounds) are subject to various regulations, since many environmental side-effects have been reported. The chemicals have the potential to volatilize or leach into the environment, where they can accumulate in fatty tissues and thus enter the food chain. In addition, the fire products from halogenated flame-retardant system are mostly both toxic and acidic, causing post-fire corrosion issues. Therefore, it is desirable that the flame retardants developed in the future have little effect on the environment. Research on reactive flame-retardant approaches that can be incorporated directly into polymer structures or the development of recyclable flame retardants is still actively underway, but more time is expected to be necessary to reach economic feasibility. In the future, more focus should be placed on the development of more environmentally friendly flame retardants. Furthermore, we believe that the development of inherently fire-resistant polymers will also attract attention. It will be highly important to identify the fire-resistant units and properly combine them with other units in developing new fire-resistant polymers.

## Figures and Tables

**Table 1:** Combustion heats of several polymers in common use. Reproduced with permission from Ref. [35]. Copyright 2016, MDPI AG.

Polymer	Heat of Combustion (Δ*H*, kJ/g)
Polyethylene	46.5
Polypropylene	46.5
Polybutadiene	45.2
Polystyrene	41.5
Acrylonitrile butadiene styrene copolymer	36.0
Polycarbonate	31.0
Poly(methyl methacrylate) (PMMA)	26.1
Poly(vinyl chloride)	24.7
Polyethylene terephthalate	22.2
Cotton	17.0
Cellulose	16.7

**Table 2:** Classification of flame retardants [72,73].

Classification by Usage		Classification by Composition		Remark
Additive	Organic	Organic	Phosphorus-based	Non-halogen
			Nitrogen-based	
	Inorganic		Phosphorus-based + halogen-based	Halogen
			Halogen-based + Br or Cl compounds	
Reactive	Vinyl group Carboxyl group	Inorganic	Metal hydroxides Boron-based Antimony-based	Non-halogen
	Hydroxyl group Epoxy group			

**Table 3:** LOIs of polymeric materials. Reprinted with permission from Ref. [50]. Copyright 1988, Elsevier.

Polymer	LOI (%)
Polyoxymethylene	15.7
Polyurethane foam	16.5
Cotton	16–17
PMMA	17.3
Polyethylene	17.4
Polystyrene	17.6–18.3
Polycarbonate	22.5
Red oak	23.0
Nylon 6	25–26
Poly(vinyl chloride)	45–49
Polytetrafluoroethylene	95.0

**Table 4:** Heat release rates (HRRs) of several polymers. Ref. [142].

Polymer	Repeat Unit Structure	HRR (W cm^−2^)
Polypropylene	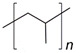	150.9
Polystyrene	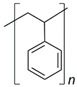	110.1
Polycarbonate	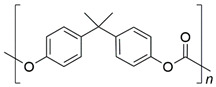	42.9
Poly(vinyl chloride)	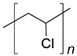	17.5
Polyethylene	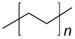	140.8
Polylactic acid	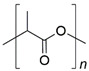	27.2

**Table 5:** UL 94 fire classifications. Reprinted with permission from Ref. [72]. Copyright 2009, Elsevier.

UL 94 V-0	*t*_1_ and *t*_2_ less than 10 s for each specimen *t*_1_ + *t*_2_ less than 50 s for the 5 specimens *t*_2_ + *t*_3_ less than 30 s for each specimen No after flame or afterglow up to the holding clamp No burning drops
UL 94 V-1	*t*_1_ and *t*_2_ less than 30 s for each specimen *t*_1_ + *t*_2_ less than 250 s for the 5 specimens *t*_2_ + *t*_3_ less than 60 s for each specimen No after flame or afterglow up to the holding clamp No burning drops
UL 94 V-2	*t*_1_ and *t*_2_ less than 30 s for each specimen *t*_1_ + *t*_2_ less than 250 s for the 5 specimens *t*_2_ + *t*_3_ less than 60 s for each specimen No after flame or afterglow up to the holding clamp Burning drops allowed

**Table 6:** Mass fractions of residues normalized by the original sample mass collected after gasification tests. Reprinted with permission from Ref. [161]. Copyright 2005, Elsevier.

Mass Fraction of SWNTs (%)	0.0	0.2	0.5 ^a^	0.5	1.0
Residual mass/original mass (%)	0.0	0.07 ± 0.05	0.76 ± 0.05	0.99 ± 0.05	1.81 ± 0.05

^a^ Poor dispersion.

**Table 7:** Cone calorimetry, LOI, and UL 94 analysis results for various samples. Reprinted with permission from Ref. [189]. Copyright 2014, Elsevier.

Sample	TTI (s)	PHRR (KW m^−2^)	ASEA (m^2^ kg^−1^)	AMLR (g s^−1^)	LOI (%)	UL 94
PP	42 ± 4	1242 ± 21	552 ± 14	0.049 ± 0.006	17.8	Failed
PP/MWNTs	44 ± 2	538 ± 12	511 ± 15	0.048 ± 0.004	20.6	Failed
PP/RGO	43 ± 3	486 ± 14	474 ± 12	0.048 ± 0.004	20.1	Failed
PP/MWNTs/RGO	48 ± 2	465 ± 12	439 ± 12	0.046 ± 0.005	21.0	Failed
PP/IFR	53 ± 4	350 ± 11	412 ± 13	0.037 ± 0.003	29.2	V-1
PP/IFR/MWNTs	64 ± 3	278 ± 11	401 ± 13	0.035 ± 0.004	29.	V-1
PP/IFR/RGO	66 ± 4	245 ± 12	396 ± 14	0.034 ± 0.004	30.6	V-1
PP/IFR/MWNTs/RGO	82 ± 3	212 ± 8	380 ± 12	0.032 ± 0.003	31.4	V-0

TTI: time of ignition; PHRR: peak heat release rate; ASEA: average specific extinction area; and AMLR: average mass loss rate.

**Table 8:** Compositions of polyamide 6 nanocomposites. Reprinted with permission from Ref. [221]. Copyright 2006, Elsevier.

Sample	Formulation (wt%)	CP Ratio (wt%)	Process
A0	PA6/CNBR = 90/40	-	-
A1	PA6/CP_A_ = 90/100	CNBR/nano-MDH = 40/60	New process (illustrated in Figure 30)
A2	PA6/CNBR/nano-MDH = 90/40/60	-	Conventional process

PA6: polyamide 6; CNBR: carboxyl acrylonitrile butadiene; CP: compound powder.

**Table 9:** Major data summarizing the fire performance for various samples.

	LOI (%)	PHRR (kW m^−2^)	THR (MJ m^−2^)	TTI (s)	SET (s)	UL 94	Ref.
Functionalized MWNTs	29.2					V-0	[176]
Epoxy/MWNTs composites	33.6	754 ± 31	102 ± 3	-		-	[181]
RGO and PP/MWNTs	31.4	212 ± 8	-	82 ± 3		V-0	[189]
MOGO and MOF	29	702 ± 9					[195]
Modified GO with nano ZIF-8	27	258 ± 7	29.9	62 ± 1	10.2	V-2	[199]
MoS_2_@TiO_2_ structure		314.25 ± 1.82	53.58 ± 0.21				[232]
Nano-MDH		277		150			[221]
ATH and MDH					7 s for ATH6 s for MDH		[85]
Nano ZIF-8/PP composites	25					V-2	[238]
Nano ZIF-8/PLA composites	26				2.9 s	V-2	[210]

LOI: limited oxygen index; PHRR: peak heat release rate; THR: total heat release; TTI: time to ignition; and SET: self-extinguishing time.
